# Design, Synthesis, and Evaluation of Novel (−)*‐cis‐N‐*Normetazocine Derivatives: In Vitro and Molecular Modeling Insights

**DOI:** 10.1111/cbdd.70037

**Published:** 2024-12-26

**Authors:** Giuliana Costanzo, Alessandro Coco, Giuseppe Cosentino, Vincenzo Patamia, Carmela Parenti, Emanuele Amata, Agostino Marrazzo, Antonio Rescifina, Lorella Pasquinucci

**Affiliations:** ^1^ Department of Drug and Health Sciences University of Catania Catania Italy

**Keywords:** ADME studies, *cis*‐*N*‐normetazocine, docking studies, mu opioid receptor, radioligand competition‐binding

## Abstract

Suitable structural modifications of the functional groups at *N*‐substituent of (−)‐*cis*‐*N*‐normetazocine nucleus modulate the affinity and activity profile of related ligands toward opioid receptors. Our research group has developed several compounds and the most interesting ligands, **LP1** and **LP2**, exhibited a dual‐target profile for mu‐opioid receptor (MOR) and delta‐opioid receptor (DOR). Recent structure–affinity relationship studies led to the discovery of novel **LP2** analogs (compounds **1** and **2**), which demonstrated high MOR affinity in the nanomolar range. Here, we reported the synthesis of the new (−)‐*cis*‐*N*‐normetazocine derivatives (**3**–**8**) characterized by the absence of the phenyl ring in the *N*‐substituent compared to all previous reported ligands. Compounds **3** and **4**, featuring a methyl ester functional group in the *N*‐substituent, retained significant MOR affinity and exhibited similar affinity for the kappa‐opioid receptor (KOR). In contrast, compounds **7** and **8**, which contain a hydroxamic acid functionality, maintained affinity exclusively toward MOR. Neither of compounds (**3**–**8**) showed DOR affinity. Molecular modeling studies confirmed a similar docking pose in the MOR binding pocket for these compounds. Additionally, the in silico ADME profile of the most interesting ligands (**3**, **4**, **7**, and **8**) was investigated revealing a favorable profile for compound **7** regarding the blood–brain barrier permeability, suggesting its potential as a peripherally restricted opioid ligand.

## Graphical abstract

New (−)‐*cis*‐*N*‐normetazocine derivatives were developed (compounds **3**–**8**) and the most interesting compound **7** showed a significant MOR affinity and a similar docking mode with respect to previously synthesized compounds. Moreover, the limited BBB permeability of compound **7** suggests its potential for development as a peripherally restricted opioid ligand.

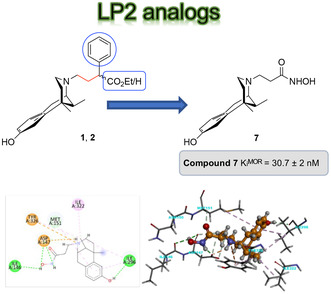

## 1 Introduction

Normetazocines with (−)‐(2*R*,6*R*,11*R*) configuration have shown high affinity toward opioid receptors, and modifications to the functional groups attached to the basic nitrogen have produced compounds with varying pharmacological profiles at MOR (mu opioid receptor), DOR (delta opioid receptor), and KOR (kappa opioid receptor) (Turnaturi, Marrazzo, et al. 2018). Our research group has synthesized and evaluated numerous (−)‐*cis*‐*N*‐normetazocine‐based compounds, focusing on *N*‐substituents that can shift their affinity, selectivity, and activity versus opioid receptors from multitarget to selective ligands (Pasquinucci et al. 2021).

**FIGURE 1:**
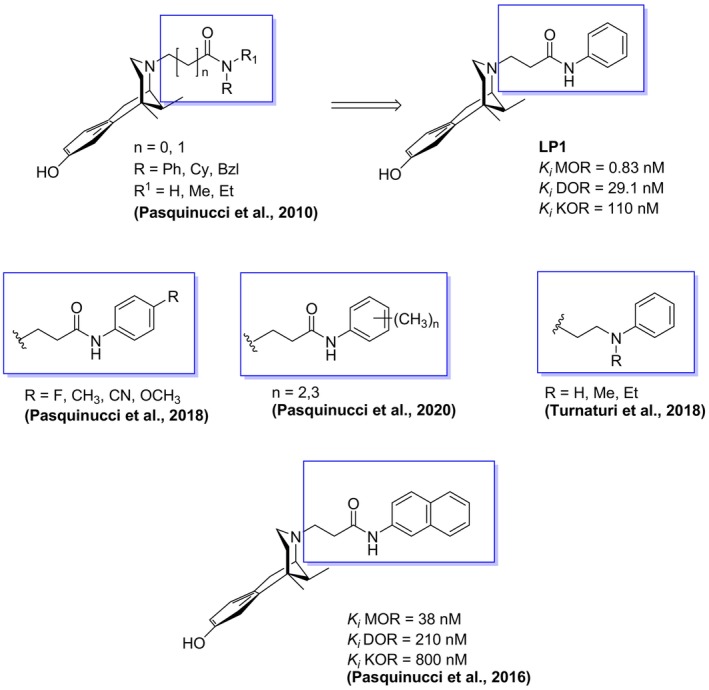
Structures of **LP1** and its analogs.

First, at the basic nitrogen of (−)‐*cis*‐*N*‐normetazocine nucleus we introduced primary or secondary amido substituents with phenyl or cyclohexyl rings. Among these synthesized compounds, the dual‐target MOR/DOR ligand **LP1**, bearing a phenylpropanamido substituent, has emerged as particularly unique (Figure 1) (Pasquinucci et al. 2010). Steric and electronic characteristics of its phenyl ring were evaluated though the introduction of electron‐donating or electron‐withdrawing groups and phenyl ring alkylation in various ways and positions obtaining different affinity and selectivity profiles of relative compounds versus opioid receptor's types (Figure 1) (Pasquinucci et al. 2018, 2020). Moreover, the replacement of its phenyl with bulkier rings shifted their MOR activity profile to antagonism and a potent MOR antagonist with a *K*_i_ of 38 nM and an antagonist affinity (pA_2_) of 8.6 nM was obtained (Figure 1) (Pasquinucci et al. 2016). Respect to amide functionality, the introduction of a secondary amide decreased MOR affinity of compounds, due to steric bulk of the alkyl amido substituents (Figure 1) (Pasquinucci et al. 2010). A critical distance between the phenylpropanamido functionality and basic nitrogen of (−)‐*cis*‐*N*‐normetazocine nucleus of **LP1** was evaluated varying the length of the spacer in its *N*‐substituent. In fact, both methylene deletion and introduction in propanamido spacer resulted in a dramatic loss of affinity for opioid receptors of relative compounds (Pasquinucci et al. 2010). However, the amide functionality substitution with more flexible ethylamino and propylamino *N*‐substituents, showing a second positive charge at the *N*‐substituent, shifted versus compounds with MOR and KOR affinity profile (Figure 1) (Turnaturi, Parenti, et al. 2018). In summary, in **LP1** derivatives, despite different electronic and steric variabilities in *N*‐substituent was tolerated versus MOR profile, DOR affinity was completely lost thus missing the dual‐target MOR/DOR profile of **LP1**.

**FIGURE 2:**
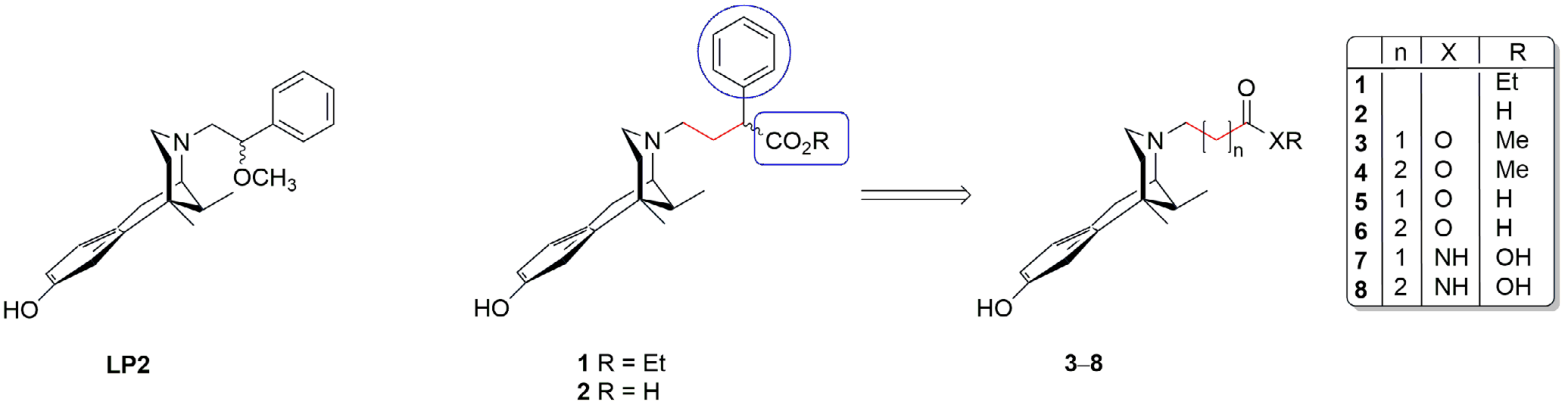
Structures of **LP2** and its analogs **1** and **2** (Costanzo et al. 2023) with the sites of modification and the novel synthesized compounds (**3**–**8**).

Shorter and flexible *N*‐substituents were introduced in a series of (−)‐*cis*‐*N*‐normetazocine‐based compounds and the (*R*/*S*)‐2‐methoxy‐2‐phenyl‐ethyl derivative, named **LP2**, maintained the opioid receptors' affinity of **LP1** compound but shifted its activity profile at DOR from antagonist to agonist (Figure 2) (Pasquinucci et al. 2017).

Both **LP1** and **LP2** compounds have shown valuable in vivo behavioral profiles, exhibiting antinociceptive effects in models of nociceptive and persistent pain (Parenti et al. 2013; Vicario et al. 2019). At the molecular level, they have demonstrated protective effects against the development of chronic neuropathic pain by inhibiting neuroinflammation (Vicario et al. 2019, 2022; Fidilio et al. 2021). Evaluation of the 2*R*‐and 2*S*‐diastereoisomers of the dual‐target MOR/DOR agonist **LP2** revealed their unique abilities to promote receptor/G‐protein and receptor/β‐arrestin 2 interactions. Notably, the (2*S*)‐**LP2** isomer preferentially activated the G‐protein pathway over β‐arrestin 2, behaving as a biased agonist at MOR and predominantly at DOR (Pasquinucci et al. 2019).

Our recent structure–affinity evaluation of **LP2** analogs identified compounds **1** and **2**, demonstrating significant MOR affinity (Figure 2) (Costanzo et al. 2023). These compounds, at *N*‐substituent, featured ethoxycarbonyl and carboxylic functional groups, respectively, and a two‐methylene unit spacer that maximized their interactions with MOR. Molecular modeling studies highlighted that compounds **1** and **2** could accommodate within the MOR binding site, optimizing interactions, and strengthening anchoring. Moreover, the influence of *N*‐substituent lipophilicity/hydrophilicity in opioid receptor interaction has been previously studied. In this regard, Metcalf et al. (2008) synthesized different compounds bearing at *N*‐substituent ester or carboxylic acid groups. The ester and carboxylic acid derivatives showed various affinities for all opioid receptors and the spacer length of the *N*‐substituent further stressed its importance for in vitro activity. In these *N*‐acid derivatives, the elongation of the spacer reduced the activity, while in the *N*‐ester series, it has been observed that a switch of activity profile from antagonism to agonism.

To further enhance our understanding of the interactions between the *N*‐substituent and the opioid receptors, we designed novel (−)‐*cis*‐*N*‐normetazocine derivatives **3**–**8** (Figure 2). In these compounds, the phenyl ring in the *N*‐substituent was removed to assess its necessity for MOR interaction. Additionally, the ester (**3** and **4**) and the carboxylic functional groups (**5** and **6**) were either maintained or replaced with a hydroxamic acid functional groups (**7** and **8**). Spacers of different lengths were also introduced at the *N‐*substituent. We evaluated the in vitro affinity and selectivity profiles of these compounds against all opioid receptors and sigma receptors through competition binding assays. Molecular modeling studies were also conducted to analyze differences in their binding mode. Moreover, the ADME profiles of the most promising compounds (**3**, **4**, **7**, and **8**) were assessed using the Swiss ADME online tool to gather information on their pharmacokinetic properties.

## 2 Results and Discussion

### 2.1 Chemistry

**SCHEME 1:**
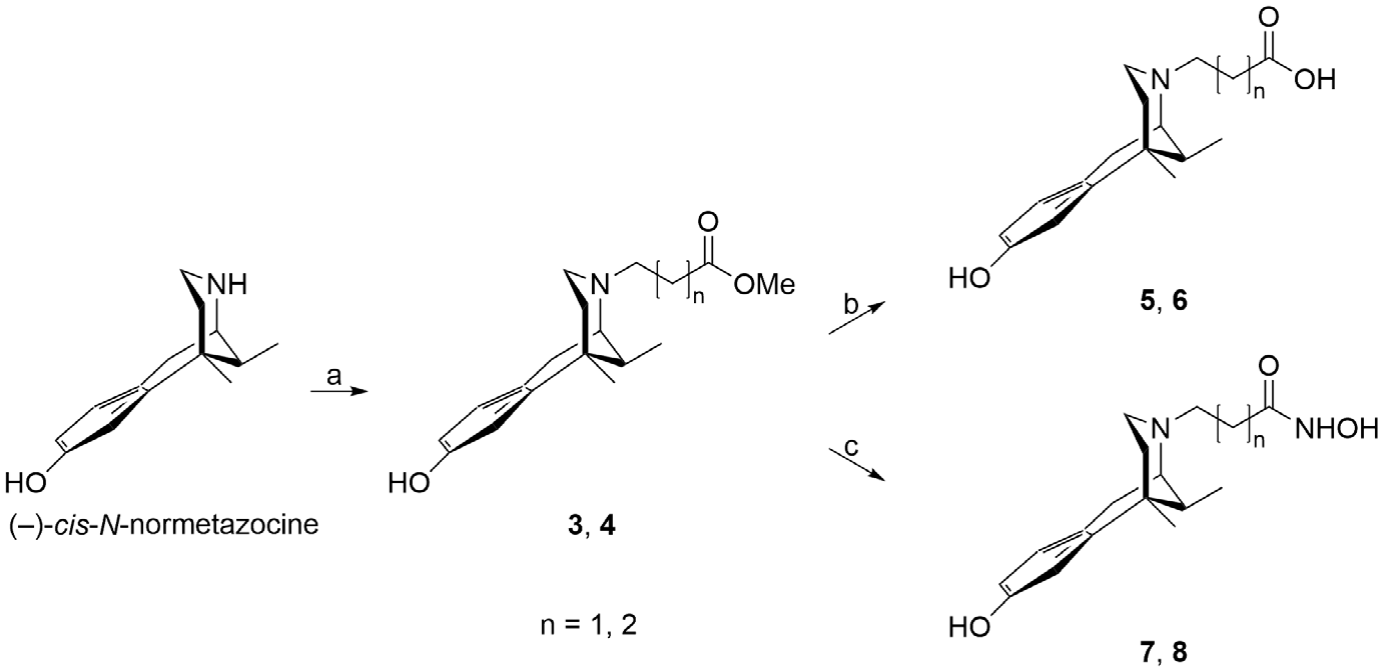
Synthesis of target compounds **3**–**8**: (a) methyl 3‐bromopropanoate or methyl 4‐chlorobutanoate, NaHCO_3_, KI, DMF, 55°C, 24 h; (b) NaOH 1 M, 110°C, 5 h; (c) NH_2_OH hydrochloride, NaOMe, BuOH, r.t., 24 h.

Compounds **3**–**8** were synthesized according to Scheme 1. Target esters **3** and **4** were obtained by alkylation of (−)‐*cis*‐*N*‐normetazocine with methyl‐3‐bromopropanoate and methyl 4‐chlorobutanoate, respectively. The ester hydrolysis under basic conditions yielded the respective acid derivatives **5** and **6**. The hydroxamic acid derivatives **7** and **8** were synthesized by treating the corresponding esters with NH_2_OH hydrochloride in the presence of NaOMe. The final compounds were characterized by ^1^H NMR, attached proton test (APT), and elemental analysis.

### 2.2 Structure–Affinity Relationship Studies

The synthesized compounds **3**–**8** were evaluated for their affinity at MOR, DOR, and KOR using a radioligand binding assay with the respective [^3^H]‐DAMGO, [^3^H]‐Delthorphin, and [^3^H]‐U69,593 radioligand (Table 1). **LP2**, DAMGO, naltrindole, and (−)‐U50,488 were included as internal controls (Turnaturi et al. 2022). Moreover, the compounds were tested at the sigma 1 receptor (σ1R) and sigma 2 receptor (σ2R) using [^3^H]‐(+)‐pentazocine and [^3^H]‐1,3‐di‐ortho‐tolyguanidine ([^3^H]–DTG) radioligands, respectively (Dichiara et al. 2023). Inhibition constant (*K*_i_) values were obtained using nonlinear regression analysis with GraphPad Prism (GraphPad Software Inc., San Diego, USA) (Figures S1 and S2).

**TABLE 1:** MOR, DOR, and KOR binding affinity and selectivity of compounds **3**–**8**.

Compound	*K*_i_ (nM) ± SD^a^				
	MOR	DOR	KOR	*K*_i_^DOR^/*K*_i_^MOR^	*K*_i_^KOR^/*K*_i_^MOR^
**1** ^b^	10.80 ± 2	440.00 ± 22	130.00 ± 15	40.70	12.00
**2** ^b^	11.80 ± 2	275.00 ± 11	3216.00 ± 60	23.30	252.70
**3**	19.01 ± 3	512.86 ± 30	60.67 ± 8	26.98	3.19
**4**	67.76 ± 7	217.77 ± 20	70.47 ± 9	3.21	1.04
**5**	382.82 ± 21	1104.08 ± 54	> 5000	2.88	—
**6**	992.26 ± 55	1204.78 ± 64	> 5000	1.21	—
**7**	30.76 ± 2	334.97 ± 19	> 5000	10.89	—
**8**	42.17 ± 3	1300.00 ± 80	> 5000	30.83	—
**LP2** ^c^	1.08 ± 0.1	6.61 ± 0.6	15.22 ± 0.8	6.11	14.10
DAMGO	1.16 ± 0.1	—	—	—	—
Naltrindole	—	1.13 ± 0.1	—	—	—
U69,593	—	—	0.34 ± 0.1	—	—

^a^ Each value is the mean ± SD of at least two experiments performed in duplicate. Reference compounds were tested with the same membrane homogenates.

^b^ Pasquinucci et al. (2017).

^c^ Costanzo et al. (2023).

Compared with compound **1**, compounds **3** and **4**, which retained an ester functionality in the *N*‐substituent despite lacking the phenyl ring, showed MOR affinity with the *K*_i_ values of 19.01 and 67.76 nM, respectively. Compound **3** exhibited higher MOR affinity than compound **4**, likely due to differences in spacer length. Moreover, compounds **3** and **4** demonstrated similar affinities for KOR, with *K*_i_ values of 60.07 and 70.5 nM, respectively. Moreover, the compounds **3** and **4** did not display a relevant affinity for DOR, similar to the reference compound **1**. Compounds **5** and **6**, containing an acid functional group, showed a loss of affinity for all opioid receptors. The absence of a phenyl ring in these compounds, as compared to compound **2**, likely inhibits their interaction with the receptor cavity, suggesting they do not meet the minimum requirements for effective binding. However, compounds **7** and **8**, which possess a hydroxamic acid functionality in the *N*‐substituent, maintained an affinity for MOR but exhibited a reduced affinity for KOR. Both compounds also showed negligible affinity for DOR. Regarding σ1R and σ2R affinities, all new compounds (**3**–**8**) displayed no significant affinity, with *K*_i_ values > 10,000 nM (data not shown).

Compared to the **LP2** lead compound, characterized by an *N*‐phenethylnormetazocine bearing a methoxy group at the benzylic carbon, we investigated how various structural modifications influence binding affinity toward all opioid receptors. All compounds **3**–**8** exhibited a reduced affinity for opioid receptors compared to **LP2**. However, compounds **3**, **4**, **7**, and **8** maintained a significant affinity for MOR, albeit losing the dual‐target MOR/DOR profile of **LP2**. Despite this, we demonstrated that the ester and hydroxamic acid functional groups in these new ligands can partially compensate for losing the phenyl ring's binding interactions with MOR. In silico molecular modeling studies were performed to further understand these interactions to rationalize ligand‐receptor interactions within the receptor site.

### 2.3 Molecular Modeling

#### 2.3.1 Docking Studies

The in silico studies were performed using the YASARA Structure suite, with the AutoDock software employing the Lamarckian Genetic Algorithm (LGA), as described in previous work (Costanzo et al. 2023). Ligands were prepared using Marvin Sketch software, which calculated all protonation states at pH 7.4 (Rescifina et al. 2014; Szczepańska et al. 2021; Varrica et al. 2018).

At physiological pH, the ligands predominantly exist in a protonated state with a positive charge on the nitrogen atom, which is crucial for interaction with opioid receptors. This protonation results in new stereocenters, leading to two possible protonation states. It has been established that the **LP2** compound aligns best with the experimental *K*_i_ in its (*R*)‐*N* form (Costanzo et al. 2023). Docking procedures were performed for all newly synthesized compounds (**3**–**8**), previously synthesized compounds (**LP2**, **1**, and **2**) used as internal references, and standards such as DAMGO, naltrindole, and U69,593. All compounds were simulated in both (*R*/*S*)‐*N* forms, with no significant differences observed between the two forms; thus, only the simulated *K*_i_ values that mostly closely fit the experimental *K*_i_ are reported (Table 2). The **LP2** compound was again shown to fit best in its (*R*)‐*N* form.

**TABLE 2:** Calculated free energies of binding, Δ*G* (kcal/mol), and constants of binding, *K*_i_ (nM), for the binding sites of MOR, DOR, and KOR for all synthesized compounds and other reference compounds.

Compound	MOR			DOR			KOR		
	Calcd ΔG (kcal/mol)	Calcd *K*_i_ (nM)	Expected *K*_i_ (nM)	Calcd ΔG (kcal/mol)	Calcd *K*_i_ (nM)	Expected *K*_i_ (nM)	Calcd ΔG (kcal/mol)	Calcd *K*_i_ (nM)	Expected *K*_i_ (nM)
**1**	−10.93	19.70	10.80 ± 2	−8.97^b^	474.53	440.00 ± 22	−10.12	73.37	130.00 ± 15
**2**	−11.01	17.30	11.80 ± 2	−9.30^b^	277.73	275.00 ± 11	−7.74^b^	3494.63	3216.00 ± 60
**3**	−10.10^a^	75.79	19.01 ± 0.45	−9.26	296.36	512.86 ± 0.54	−9.55^a^	185.09	60.67 ± 0.7
**4**	−9.77^a^	129.50	67.76 ± 0.52	−9.86	111.90	217.77 ± 0.65	−9.29	282.27	70.47 ± 0.76
**5**	−8.66	784.90	382.82 ± 0.63	−8.47	1068.47	1104.08 ± 0.75	−7.42^c^	5874.86	> 5.000
**6**	−8.87	558.17	992.26 ± 0.83	−9.11	378.07	1204.78 ± 55	−7.63^c^	4177.82	> 5.000
**7**	−9.89^a^	106.58	30.76 ± 0.5	−9.70	145.09	334.97 ± 45	−8.52^c^	985.17	> 5.000
**8**	−10.06	80.88	42.17 ± 0.86	−8.52^b^	985.17	1300.00 ± 0.75	−7.47^b^, ^c^	5416.86	> 5.000
**LP2**	−10.67^a^	30.05	1.08 ± 0.10	−10.20	64.44	6.61 ± 0.60	−10.58	34.77	15.22 ± 0.80
DAMGO	−12.22	2.43	1.16 ± 0.10	—	—	—	—	—	—
Naltrindole	—	—	—	−11.02	17.02	1.13 ± 0.10	—	—	—
U69,593	—	—	—	—	—	—	−10.62	32.59	0.34 ± 0.10

^a^ The docking score and so the simulated *K*_i_ were adjusted by performing an energy minimization and short MD for the best docking pose. Thus, re‐docking the ligand to the receptor.

^b^ In these cases, the binding pose was selected to represent the experimental *K*_i_ and align with the common interactions.

^c^ These specific molecules don't show common interactions for the receptor, thus it is highly predictable a non‐binding interaction.

**FIGURE 3:**
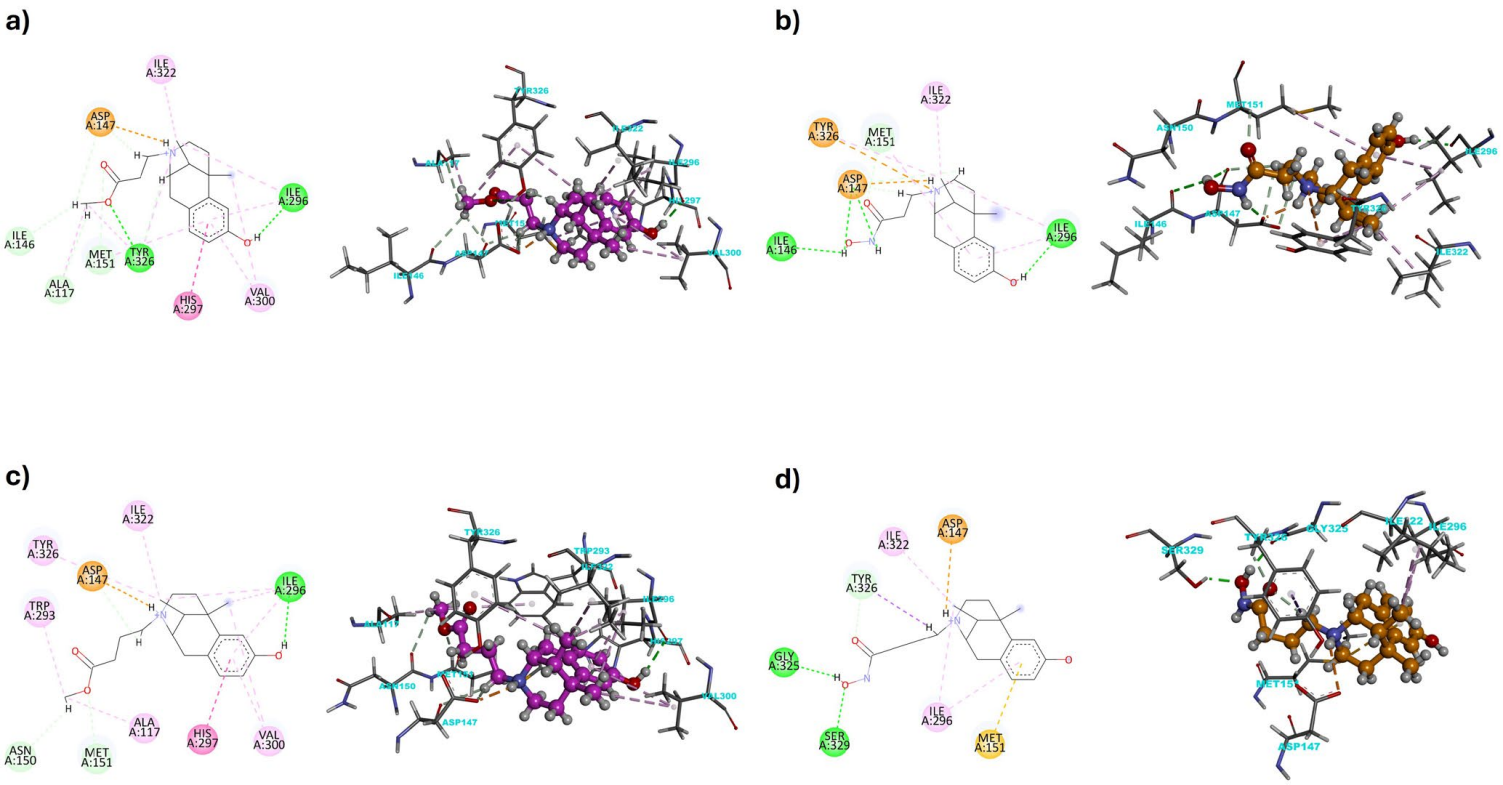
Docking simulations 2D and 3D diagrams for newly synthesized compounds with MOR receptor (PDB ID: 5C1M). (a) **3**@MOR; (b) **7**@MOR; (c) **4**@MOR; (d) **8**@MOR. Colors represent the type of interaction: Hydrophobic (light pink and light green), π (pink and purple), hydrogen bond (green), and salt bridge (orange).

Considering MOR, the most interesting compounds are **3**, **4**, **7**, and **8** (Table 2), with experimental *K*_i_ values ranging from 19 to 68 nM, and calculated Δ*G* values of −10.10, −9.77, −9.89, and − 10.06 kcal/mol, respectively. All four compounds form the classic salt bridge between the positively charged nitrogen and Asp147. Compounds **3**, **4**, and **7** also share a hydrogen bond with the hydroxyl group on the aromatic ring, interacting with Ile296, increasing the complex's stability. Compound **8** lacks this specific interaction due to its longer chain but achieves better accommodation with Ile296 through hydrophobic interactions while forming two new hydrogen bonds with the hydroxamic acid, Gly325, and Ser329 (Figure 3). Additional interactions include hydrogen bonds with Tyr326 for compound **3** and Ile146 for compound **7**. Notably, there is a π‐π T‐shaped interaction with His297 for both compounds **3** and **4**, a π‐cation interaction with Tyr326 for compound **7**, and a π‐sulfur interaction with Met151 for compound **8**. These poses are further stabilized by a network of hydrophobic interactions involving Ile322 for all compounds and additional residues such as Val300, Ala117, and Met151 for compounds **3** and **4** and Met151 and Ile296 for compounds **7** and **8**, respectively.

**FIGURE 4:**
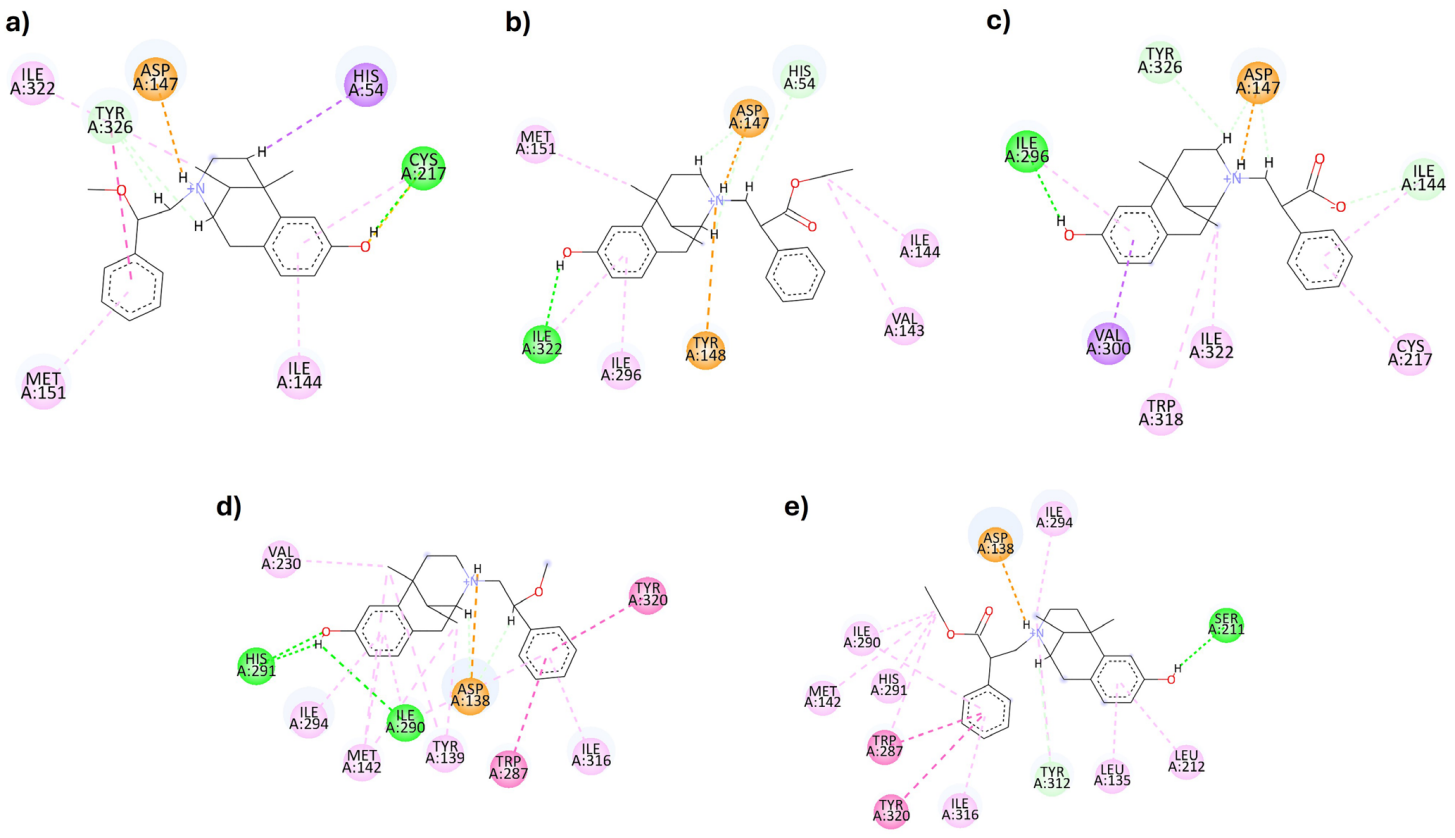
Docking simulations 2D diagrams for preceding synthesized compounds with MOR (PDB ID: 5C1M) and KOR (PDB ID: 4DJH) receptors as reference. (a) **LP2**@MOR; (b) **1**@MOR; (c) **2**@MOR; (d) **LP2**@KOR; (e) **1**@KOR. Colors represent the type of interaction: Hydrophobic (light pink and light green), π (pink and purple), hydrogen bond (green), and salt bridge (orange).

The newly synthesized ligands lack the phenyl ring compared to compounds **1** and **2** (Figure 4). This structural difference impacts their accommodation within the binding pocket, enhancing flexibility while reducing hydrophobic interactions. The increased flexibility does not benefit compounds **5** and **6**, which tend to clash with the key acid residue Asp147 due to the carboxylic acidic portion.

**FIGURE 5:**
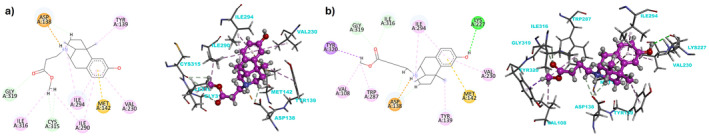
Docking simulations 2D and 3D diagrams for newly synthesized compounds with KOR receptor (PDB ID: 4DJH). (a) **3**@KOR; (b) **4**@KOR. Colors represent the type of interaction: Hydrophobic (light pink and light green), π (pink and purple), hydrogen bond (green), and salt bridge (orange).

KOR, with Δ*G* values of −9.55 and − 9.29 kcal/mol, respectively (Table 2). Compared to **LP2** and compound **1**, which also display good inhibitory activity against KOR, the newly synthesized compounds reveal that carboxylic acid and hydroxamic acid functionalities are not well‐tolerated in the KOR binding pocket. Conversely, the presence of only the ester portion in compounds **3** and **4**, as opposed to both the ester and phenyl moieties in compound **1**, slightly increases affinity by reducing the total molecular volume. Both compounds form a salt bridge between the positively charged nitrogen and Asp138, a characteristic feature of KOR ligands (Figure 5). They also engage an extensive network of hydrophobic interactions, including Tyr139, Val230, and Ile294, similar to **LP2** and compound **1** (Figure 4). Additionally, a π‐sulfur interaction with Met142 characterizes both compounds. Compound **4** is further stabilized by a π‐σ with Tyr320 and a hydrogen bond with Lys227. Compound **3** shows slightly better affinity, likely due to its shorter chain, which allows for a better fit within the binding pocket.

#### 2.3.2 Molecular Dynamics (MD) Simulations

**FIGURE 6:**
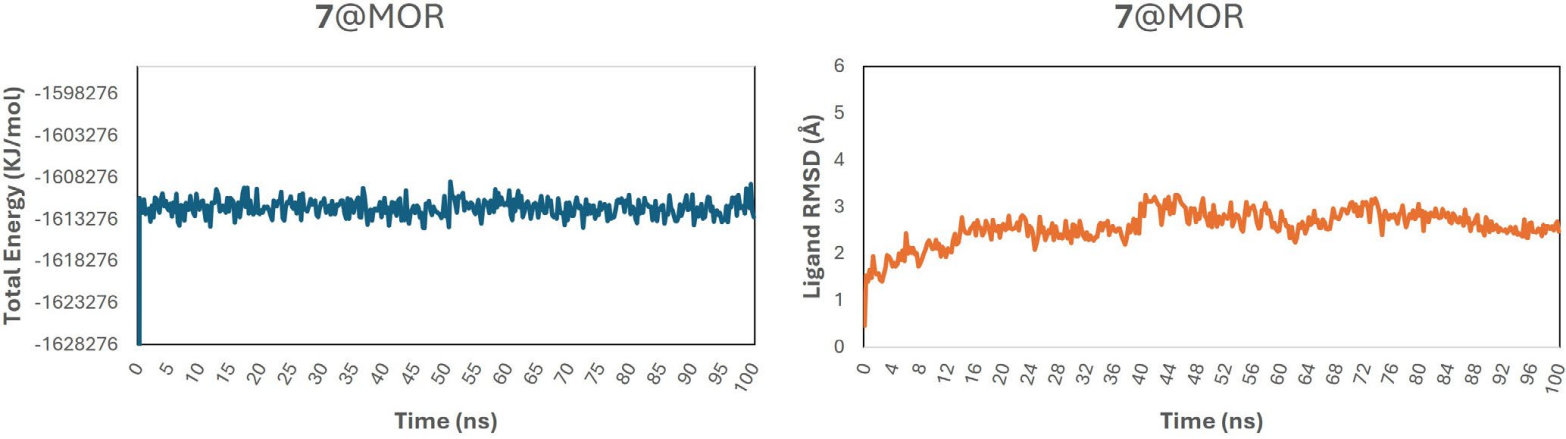
Total energy and ligand RMSD for **7**@MOR complex.

Given the notable high affinity and selectivity of compound **7** for the MOR (calculated Δ*G* − 9.89 kcal/mol), we conducted an in‐depth study of the **7**@MOR complex through a 100 ns MD simulation. The total energy graph analysis reveals that the complex achieves equilibrium after 250 ps. The ligand RMSD indicates an average fluctuation of approximately 1.5 Å (Figure 6), demonstrating that the molecule consistently remains within the binding site throughout the simulation. This stability is attributed to strong interactions, a persistent salt bridge, and the same hydrogen bonds maintained throughout the MD simulation. Additionally, the hydrophobic network further anchors the ligand within the binding site (Gentile et al. 2022).

### 2.4 In Silico ADME Profile

Investigating absorption, distribution, metabolism, and excretion (ADME) properties is crucial in drug development. Various parameters related to drug pharmacokinetics, such as octanol–water partition coefficient, water solubility, intestinal absorption, and bioavailability, play significant roles (Zhu et al. 2018). Numerous in silico models are available to predict the ADME properties of drug candidates. In this study, the ADME profiles of the most promising compounds were assessed using the free web tool Swiss ADME (Daina, Michielin, and Zoete 2017). The computed ADME properties are presented in Table 3. The drug‐likeness of the novel ligands was evaluated based on Lipinski's rules of five, which states that a compound is likely to be a good drug candidate if it meets at least two of the following criteria: MW < 500, LogP ≤ 5, HBA ≤ 10, HBD ≤ 5, TPSA < 40 Å^2^ (Chen et al. 2020). The prediction indicated that all ligands complied with Lipinski's rules of five, showing zero violation. The results revealed that compounds **3**, **7**, and **8** were water‐soluble, while compound **4** was moderately water‐soluble. Additionally, the ability of these compounds to cross the blood–brain barrier (BBB) was evaluated.

**TABLE 3:** In silico ADME profile of compounds **3**, **4**, **7**, and **8**.

Compound	LogP_o/w_	Water‐solubility	BBB permeability	GI absorption	CYP inhibition	Lipinski's rule
**3**	2.97	Soluble	Yes	High	CYP2D6	0 violations
**4**	3.15	Moderate	Yes	High	CYP2D6	0 violations
**7**	1.94	Soluble	No	High	No	0 violations
**8**	1.92	Soluble	Yes	High	CYP2D6	0 violations

**FIGURE 7:**
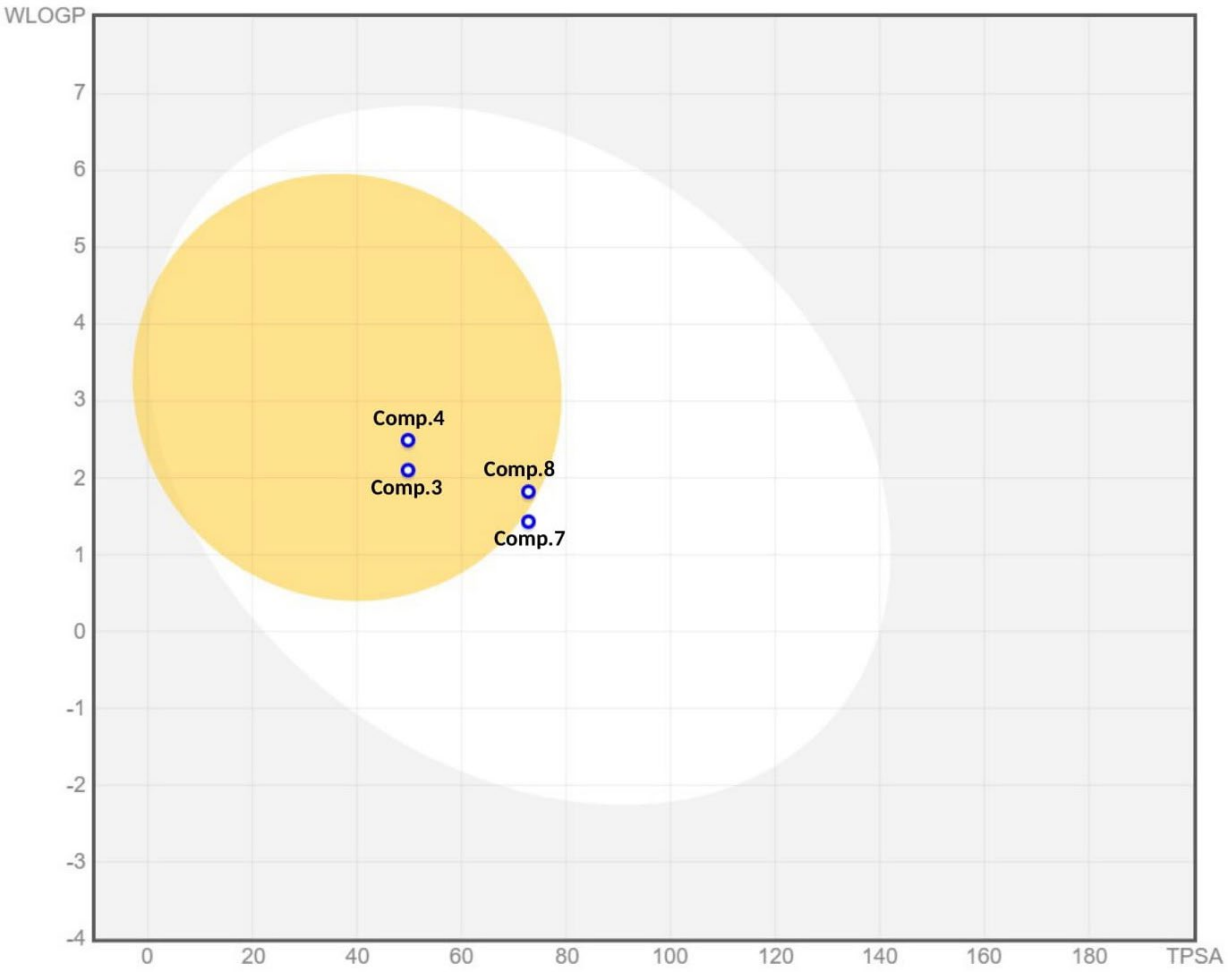
BOILED‐Egg model: The yellow sphere represents the brain permeation region, and the oval white part represents the gastrointestinal absorption region. The grey area represents the minimal absorption and partial brain permeation region. The molecule is designed as a blue sphere. The sphere is colored blue if predicted as actively effluxes by P‐gp (PGP+) and red if predicted as non‐substrate of P‐gp (PGP−).

The BBB permeability values of compounds **3**, **4**, and **8** indicated that these ligands could readily penetrate the central nervous system. Conversely, compound **7** was predicted not to cross the BBB, likely due to its lower lipophilicity. This is illustrated by the BOILED‐Egg model, which intuitively evaluates passive gastrointestinal absorption (HIA) and BBB penetration (Figure 7). Moreover, all compounds showed potential to be substrates of P‐glycoprotein (P‐gp). The predicted gastrointestinal permeability was high for all selected compounds. Regarding the metabolism parameters, the inhibition of the main cytochromes (CYP1A2, CYP2C19, CYP2C9, CYP2D6, and CYP3A4) was predicted in a simple binary form (Yes/No). The results indicated that compounds **3**, **4**, and **8** could be CYP2D6 inhibitors, while compound **7** was expected to show no inhibitory activity against any of the CYP isoforms.

## 3 Conclusion

Novel (−)‐*cis*‐*N*‐normetazocine derivatives **3**–**8**, lacking the phenyl ring in the *N*‐substituent, were designed and synthesized. In vitro competition binding assays revealed that compounds **3**, **4**, and **7**, **8**, featuring methyl ester and hydroxamic acid functionalities, respectively, maintained significant MOR affinity. Molecular modeling studies confirmed a similar docking pose within the MOR binding pocket for these compounds. Moreover, the favorable ADME profile of compound **7**, thanks to its limited BBB permeability, suggests its potential for development as a peripherally restricted opioid ligand. This approach involves designing hydrophilic compounds to reduce BBB penetration, and compound **7**, with its hydroxamic acid functionality and shorter *N*‐substituent spacer, appears to fit this profile. Since opioid receptor expression levels in peripheral neurons are upregulated under inflammatory conditions, developing ligands restricted to the peripheral nervous system could be highly beneficial. Achieving peripheral opioid analgesia with fewer side effects‐associated respect to agonists that activate receptors in the central nervous system is desirable in inflammatory pain management (Janson and Stein 2003).

## 4 Experimental Section

### 4.1 Chemistry

#### 4.1.1 General Remarks

Reagent‐grade chemicals were purchased from Merck (Darmstadt, Germany) and were employed without further purification. (−)‐*cis*‐*N*‐normetazocine was obtained as previously reported (Brine et al. 1990). Flash column chromatography was carried out on Merck silica gel 60 (230–400 mesh). Reactions were checked by thin‐layer chromatography (TLC) performed on 250 μm silica gel Merck60 F254 coated aluminum plates; the spots were visualized by UV light or iodine chamber. Melting points were determined in open capillary tubes with a Büchi 530 apparatus and were uncorrected. 1H NMR and attached proton test (APT) spectra were recorded at 200 and 500 MHz on Varian Inova spectrometers in CDCl_3_ and DMSO‐*d*_6_. Chemical shifts *δ* are expressed in parts per million (ppm). The following abbreviations designate the multiplicities: s = singlet, d = doublet, t = triplet, m = multiplet. Elemental analyses (C, H, N) were performed on a Carlo Erba 1106 analyzer, and the results were within ±0.4% of the theoretical values.

#### 4.1.2 General Procedure for the Synthesis of the Target Compounds **3**–**8**

##### 4.1.2.1 *Methyl 3‐[(2R,6R,11R)‐8‐hydroxy‐6,11‐dimethyl‐1,4,5, 6‐tetrahydro‐2,6‐methanobenzo[d]azocin‐3(2H]‐yl)propanoate* (***3***)

(−)‐*cis*‐*N*‐normetazocine (1.38 mmol, 1 eq.) was dissolved in DMF (5 mL), and methyl 3‐bromopropionate (1.38 mmol, 1 eq.), NaHCO_3_ (2.07 mmol, 1.5 eq.) and KI (catalytic quantity) were added. The reaction mixture was stirred overnight at 55°C. At the reaction mixture, H_2_O (50 mL) was added, and the aqueous phase was extracted with EtOAc; the organic phase was dried over Na_2_SO_4_ and concentrated in vacuo. The crude was purified by flash chromatography (CH_2_Cl_2_/MeOH 93:7) to give a white solid. Yield: 73% (305 mg); [*α*]_D_^25^ = −57.3 (*c* = 0.1 in ethanol); Mp: 140°C–143°C; TLC CH_2_Cl_2_/MeOH (93:7 *v*/*v*): *R*_f_ = 0.48; ^1^HNMR (200 MHz, CDCl_3_, 36°C, TMS): *δ* = 6.82 (d, 1H, *J* = 8.0 Hz, Ar–H), 6.61 (s, 1H, Ar–H), 6.53 (d, 1H, *J* = 8.0 Hz, Ar–H), 3.55 (s, 3H, O–CH_3_), 2.96–2.88 (m, 4H, CH_2_, CH_2_), 2.80–2.55 (m, 4H, CH_2_, CH_2_), 2.18–2.07 (m, 1H, CH), 1.88–1.79 (m, 2H, CH_2_), 1.41–1.26 (m, 1H, CH), 1.19 (s, 3H, CH_3_), 0.74 (d, 3H, *J* = 8.0 Hz, CH_3_). The –OH signal is missing (Figure S3). APT (50 MHz, DMSO‐*d*_6_, 36°C, TMS): *δ* = 174.63 (Ar–C), 166.33 (Ar–C), 155.67 (Ar–C), 141.26 (Ar–C), 127.62 (Ar–C), 124.96 (Ar–C), 113.21 (CH_2_), 111.72 (CH_2_), 57.11 (CCH), 54.06 (CH_3_), 44.26 (CCH), 40.81 (CH_2_), 35.88 (CH_2_), 34.54 (CH_2_), 25.30 (CH_2_), 23.43 (CH_2_), 21.46 (CH_3_),13.94 (CH_3_) (Figure S4). Anal. calcd. for C_18_H_25_NO_3_: C 71.26, H 8.31, N 4.62. Found: C 71.35, H 8.02, N 4.51 (Table S1). CAS Registry Number: 51986‐19‐7.

##### 4.1.2.2 *Methyl 4‐((2R,6R,11R)‐8‐hydroxy‐6,11‐dimethyl‐1,4,5,6‐tetrahydro‐2,6‐ methanobenzo[d]azocin‐3(2H)‐yl)butanoate* (***4***)

(−)‐*cis*‐*N*‐normetazocine (1.38 mmol, 1 eq.) was dissolved in DMF (5 mL), and methyl 4‐chlorobutyrate (1.38 mmol, 1 eq.), NaHCO_3_ (2.07 mmol, 1.5 eq.) and KI (catalytic quantity) were added. The reaction mixture was stirred overnight at 55°C. At the reaction mixture, H_2_O (50 mL) was added, and the aqueous phase was extracted with EtOAc; the organic phase was dried over Na_2_SO_4_ and concentrated in vacuo. The crude was purified by flash chromatography (CH_2_Cl_2_/MeOH 93:7) to give a white solid. Yield: 72% (315 mg); [*α*]_D_^25^ = −54.5 (*c* = 0.1 in ethanol); Mp: 143°C–146°C; TLC CH_2_Cl_2_/MeOH (93:7 *v/v*): *R*_f_ = 0.50; ^1^HNMR (200 MHz, CDCl_3_, 36°C, TMS): *δ* = 6.82 (d, 1H, *J* = 10.0 Hz, Ar–H), 6.60 (s, 1H, Ar–H), 6.52 (d, 1H, *J* = 10.0 Hz, Ar–H), 3.59 (s, 3H, O–CH_3_), 3.03–2.91 (m, 1H, CH_2_), 2.88–2.78 (m, 4H, CH_2_, CH_2_), 2.12–2.06 (m, 1H, CH_2_), 2.25–2.18 (m, 2H, CH_2_), 2.12–2.06 (m, 1H, CH), 1.91–1.83 (m, 4H, CH_2_, CH_2_), 1.23–1.16 (m, 1H, CH), 1.12 (s, 3H, CH_3_), 0.75 (d, 3H, *J* = 10.0 Hz, CH_3_). The –OH signal is missing (Figure S5). APT (50 MHz, DMSO‐*d*_6_, 36°C, TMS): *δ* = 174.65 (Ar–C), 166.34 (Ar–C), 155.68 (Ar–C), 141.27 (Ar–C), 127.64 (Ar–C), 124.97 (Ar–C), 113.22 (CH_2_), 111.73 (CH_2_), 66.53 (CH_2_), 57.12 (CCH), 54.07 (CH_3_), 44.27 (CCH), 40.82 (CH_2_), 35.89 (CH_2_), 34.55 (CH_2_), 25.31 (CH_2_), 23.44 (CH_2_), 21.47 (CH_3_), 13.95 (CH_3_) (Figure S6). Anal. calcd. for. C_19_H_27_NO_3_: C 71.89, H 8.57, N 4.41. Found: C 71.64, H 8.29, N 4.32 (Table S1).

##### 4.1.2.3 *3‐((2R,6R,11R)‐8‐Hydroxy‐6,11‐dimethyl‐1,4,5,6‐tetrahydro‐2,6‐methanobenzo[d]azocin‐3(2H)‐yl)propanoic acid* (***5***)

1 N NaOH solution (12.80 mmol, 11.3 eq.) was added to methyl 3‐((2*R*,6*R*,11*R*)‐8‐hydroxy‐6,11‐dimethyl‐1,4,5,6‐tetrahydro‐2,6‐methanobenzo[*d*]azocin‐3(2*H*)yl)‐propanoate (**3**) (1.13 mmol, 1 eq.). The obtained suspension was vigorously stirred and refluxed at 110°C for 5 h. After, the mixture was cooled at rt., transferred to a separatory funnel, and partitioned (CHCl_3_/H_2_O). A 1 N solution of HCl was added to the aqueous phase to pH 5–6. A white precipitate was obtained that was separated from the aqueous phase by vacuum filtration. Yield: 74% (242 mg); [*α*]_D_^25^ = −47.5 (*c* = 0.1 in ethanol); Mp: 202°C; TLC CH_2_Cl_2_/MeOH (95:5 *v*/*v*) *R*_f_ = 0.27; ^1^HNMR (500 MHz, DMSO‐d_6_, 36°C, TMS): *δ* = 6.83 (d, 1H, *J* = 10.0 Hz, Ar–H), 6.61 (d, 1H, *J* = 5.0 Hz, Ar–H), 6.51 (dd, 1H, *J* = 10.0, 5.0 Hz, Ar–H), 2.81–2.77 (m, 2H, CH_2_), 2.66–2.60 (m, 4H, CH_2_, CH_2_), 2.43–2.40 (m, 1H, CH), 2.07–2.06 (m, 2H, CH_2_), 1.92–1.88 (m, 1H, CH_2_), 1.71–1.61 (m, 1H, CH_2_), 1.23 (s, 3H, CH_3_), 1.16–1.18 (m, 1H, CH), 0.73 (d, 3H, *J* = 10.0 Hz, CH_3_). The –OH signal is missing (Figure S7). APT (125 MHz, DMSO‐*d*_6_, 36°C, TMS): *δ* = 171.16 (Ar–C), 156.17 (Ar–C), 140.00 (Ar–C), 128.36 (Ar–C), 123.14 (Ar–C), 113.74 (Ar–C), 111.78 (CH_2_), 63.54 (CCH), 58.06 (CH_2_), 48.7 (CH_2_), 44.98 (CCH), 35.88 (CH_2_), 34.74 (CH_2_), 33.62 (CH_2_), 29.07 (CH_2_), 24.24 (CH_3_), 13.00 (CH_3_) (Figure S8). Anal. calcd. for C_17_H_23_NO_3_: C 70.56, H 8.01, N 4.84. Found: C 70.82, H 8.28, N 4.61 (Table S1).

##### 4.1.2.4 *4‐((2R,6R,11R)‐8‐Hydroxy‐6,11‐dimethyl‐1,4,5,6‐tetrahydro‐2,6‐methanobenzo[d]azocin‐3(2H)‐yl)butanoic acid* (***6***)

1 N NaOH solution (4.05 mmol, 11.3 eq.) was added to methyl 4‐((2*R*,6*R*,11*R*)‐8‐hydroxy‐6,11‐dimethyl‐1,4,5,6‐tetrahydro‐2,6‐methanobenzo[*d*]azocin‐3(2*H*)yl)‐butanoate (**4**) (0.36 mmol, 1 eq.). The obtained suspension was vigorously stirred and refluxed at 110°C for 5 h. Afterward, the mixture was cooled at r.t., transferred to a separatory funnel, and partitioned (CHCl_3_/H_2_O). A 1 N solution of HCl was added to the aqueous phase to pH 5–6. A white precipitate was obtained that was separated from the aqueous phase by vacuum filtration. Yield: 92% (100 mg); [*α*]_D_^25^ = −45.7 (*c* = 0.1 in ethanol); Mp: 197°C; TLC CH_2_Cl_2_/MeOH (95:5 *v*/*v*) *R*_f_ = 0.29; ^1^HNMR (500 MHz, DMSO‐*d*_6_, 36°C, TMS): *δ* = 7.10 (d, 1H, *J* = 4.0 Hz, Ar–H), 6.96 (d, 1H, *J* = 2.0 Hz, Ar–H), 6.78 (dd, 1H, *J* = 4.0, 2.0 Hz, Ar–H), 3.39–3.35 (m,1H, CH_2_); 3.09–3.06 (m, 1H, CH_2_), 2.96–2.89 (m, 4H, CH_2_, CH_2_), 2.37–2.34 (m, 1H, CH), 2.17–2.16 (m, 2H, CH_2_), 1.98–1.93 (m, 4H, CH_2_, CH_2_), 1.60–1.51 (m, 1H, CH), 1.47 (s, 3H, CH_3_), 0.97 (d, 3H, *J* = 4.0 Hz, CH_3_). The –OH signal is missing (Figure S9). APT (125 MHz, DMSO‐*d*_6_, 36°C, TMS): *δ* = 173.44 (Ar–C), 165.14 (Ar–C), 154.48 (Ar–C), 140.07 (Ar–C), 126.43 (Ar–C), 123.77 (Ar–C), 112.02 (CH_2_), 110.52 (CH_2_), 55.92 (CCH), 52.87 (CH_2_), 43.07 (CCH), 39.62 (CH_2_), 34.89 (CH_2_), 33.35 (CH_2_), 24.11 (CH_2_), 22.24 (CH_2_), 20.27 (CH_3_), 12.75 (CH_3_) (Figure S10). Anal. calcd. for C_18_H_25_NO_3_: C 71.26, H 8.31, N 4.62. Found: C 71.19, H 8.40, N 4.50 (Table S1).

##### 4.1.2.5 *N‐Hydroxy‐3‐((2R,6R,11R)‐8‐hydroxy‐6,11‐dimethyl‐1,4,5,6‐tetrahydro‐2,6‐methanobenzo[d]azocin‐3(2H)‐yl)propanamide* (***7***)

NH_2_OH hydrochloride (1.17 mmol, 2 eq.) was dissolved in BuOH (5 mL), and NaOMe (2.93 mmol, 5 eq.) was added and the mixture was stirred for 15 min. at rt. After that, the mixture was filtered and methyl 3‐((2*R*,6R,11*R*)‐8‐hydroxy‐6,11‐dimethyl‐1,4,5,6‐tetrahydro‐2,6‐methanobenzo[*d*]azocin‐3(2*H*)yl)‐propanoate (**3**) (0.59 mmol, 1 eq.) was added. The reaction mixture was stirred overnight at r.t. The solvent was removed in vacuo, and the crude was dissolved in H_2_O (5 mL). A 1 N solution of HCl was added to pH 4–5. The water was removed in vacuo. The crude was purified by crystallization (THF/diethyl ether) to yield a white solid. Yield: 78% (140 mg); [*α*]_D_^25^ = −42.3 (*c* = 0.1 in ethanol); Mp: 146°C–148°C; TLC CH_2_Cl_2_/MeOH (90:10 *v*/*v*) *R*_f_ = 0.18; ^1^HNMR (500 MHz, DMSO‐*d*_6_, 36°C, TMS): *δ* = 8.21 (s, 1H, NH), 6.85 (d, 1H, *J* = 10.0 Hz, Ar–H), 6.61 (s, 1H, Ar–H), 6.52 (d, 1H, *J* = 10.0 Hz, Ar–H), 3.09–3.06 (m, 1H, CH_2_), 2.86–2.80 (m, 3H, CH, CH_2_), 2.67–2.66 (m, 2H, CH_2_), 2.46–2.41 (m, 2H, CH_2_), 2.07 (m, 2H, CH_2_), 1.78–1.63 (m, 2H, CH, CH_2_), 1.22 (s, 3H, CH_3_), 0.73 (d, 3H, *J* = 5.0 Hz, CH_3_). The –OH signal is missing (Figure S11). APT (125 MHz, DMSO‐*d*_6_, 36°C, TMS): *δ* = 171.14 (Ar–C), 156.01 (Ar–C), 139.84 (Ar–C), 128.20 (Ar–C), 122.98 (Ar–C), 113.58 (Ar–C), 111.62 (CH_2_), 63.38 (CCH), 57.90 (CH_2_), 48.11 (CH_2_), 44.82 (CCH), 35.72 (CH_2_), 34.58 (CH_2_), 33.46 (CH_2_), 28.91 (CH_2_), 24.08 (CH_3_), 12.94 (CH_3_) (Figure S12). Anal. calcd. for C_17_H_24_N_2_O_3_: C 67.08, H 7.95, N 9.20. Found: C 67.10, H 8.03, N 9.37 (Table S1).

##### 4.1.2.6 *N‐Hydroxy‐4‐((2R,6R,11R)‐8‐hydroxy‐6,11‐dimethyl‐1,4,5,6‐tetrahydro‐2,6‐methanobenzo[d]azocin‐3(2H)‐yl)butanamide* (***8***)

NH_2_OH hydrochloride (0.40 mmol, 2 eq.) was dissolved in BuOH (5 mL), and NaOMe (1.00 mmol, 5 eq.) was added and the mixture was stirred for 15 min. at r.t. After that, the mixture was filtered and methyl 4‐((2*R*,6*R*,11*R*)‐8‐hydroxy‐6,11‐dimethyl‐1,4,5,6‐tetrahydro‐2,6‐methanobenzo[*d*]azocin‐3(2*H*)yl)‐butanoate (**4**) (0.19 mmol, 1 eq.) was added. The reaction mixture was stirred overnight at r.t. The solvent was removed in vacuo and the crude was dissolved in H_2_O (5 mL). A 1 N solution of HCl was added to pH 4–5. The water was removed in vacuo. The crude was purified by crystallization (THF/diethyl ether) to yield a white solid. Yield: 80% (48 mg); [*α*]_D_^25^ = −40.4 (*c* = 0.1 in ethanol); Mp: 150°C; TLC CH_2_Cl_2_/MeOH (90:10 *v*/*v*) *R*_f_ = 0.19; ^1^HNMR (500 MHz, DMSO‐*d*_6_, 36°C, TMS): *δ* = 8.45 (s, 1H, NH), 6.86 (d, 1H, *J* = 5 Hz, Ar–H), 6.63 (s, 1H, Ar–H), 6.54 (d, 1H, *J* = 10 Hz, Ar–H), 3.02–3.00 (m, 2H, CH_2_), 2.81–2.77 (m, 2H, CH_2_), 2.66–2.63 (m, 3H, CH_2_), 2.49 (m, 5H, CH, CH_2_, CH_2_), 2.24 (m, 2H, CH_2_), 2.03 (m,1H, CH), 1.25 (s, 3H, CH_3_), 0.75 (d, 3H, *J* = 5.0 Hz, CH_3_). The –OH signal is missing (Figure S13). APT (125 MHz, DMSO‐*d*_6_, 36°C, TMS): *δ* = 174.64 (Ar–C), 166.34 (Ar–C), 155.68 (Ar–C), 141.27 (Ar–C), 127.63 (Ar–C), 124.97 (Ar–C), 113.22 (CH_2_), 111.73 (CH_2_), 57.12 (CCH), 54.07 (CH_2_), 44.27 (CCH), 40.82 (CH_2_), 35.89 (CH_2_), 34.55 (CH_2_), 25.31 (CH_2_), 23.44 (CH_2_), 21.47 (CH_3_), 13.95 (CH_3_) (Figure S14). Anal. calcd. for C_18_H_26_N_2_O_3_: C 67.90, H 8.23, N 8.80. Found: C 67.67, H 8.47, N 8.85 (Table S1).

### 4.2 Radioligand Binding Assays

#### 4.2.1 Radioligand Binding Assays for Opioid Receptors

The radioligand binding assays and the data analysis were performed as previously reported (Turnaturi et al. 2022).

#### 4.2.2 Radioligand Binding Assays for σ1R and σ2R

The radioligand binding assays and the data analysis were performed as previously reported (Dichiara et al. 2023).

### 4.3 Molecular Modeling

#### 4.3.1 Structures Preparation and Minimization

All the molecules used in this study were built using Marvin Sketch (18.24. ChemAxon Ltd.). The PM6‐D3H4 Hamiltonian, implemented in the MOPAC package (MOPAC2016 v. 18.151. Stewart Computational Chemistry. Colorado Springs), was then used to further optimize the 3D structures before the alignment for the docking calculations.

#### 4.3.2 Docking Studies

Flexible ligands docking experiments were performed employing AutoDock implemented in YASARA (v. 23.12.24. YASARA Biosciences GmbH. Vienna. Austria) using the Lamarckian Genetic Algorithm (LGA). The crystal structure of MOR (PDB ID: 5C1M) (Huang et al. 2015), DOR (PDB ID: 4EJ4) (Granier et al. 2012), and KOR (PDB ID: 4DJH) (Wu et al. 2012) were retrieved from the PDB_REDO Data Bank (https://pdb‐redo.eu/). The simulation cell was generated through AutoGrid (4.2.5.1) implemented in the software with a spacing of 0.375 Å and boundaries extending 5 Å (Duan et al. 2003) from the ligand surface as reported previously (Costanzo et al. 2023). Charge points were assigned using the AMBER14 force field, the rest of the parameters remained as default, and 100 runs per ligand were performed using the “dock_runscreening” macro implemented in YASARA.

#### 4.3.3 Re‐Docking Procedure

According to the literature (Gentile, Floresta, et al. 2020; Floresta et al. 2020; De Luca et al. 2018; Gentile, Patamia, et al. 2020) the re‐docking procedure was performed by YASARA software. The complexes were subjected to an energy minimization experiment, 5 ns of MD simulation, and the average pose of the last 3 ns was selected. The resulting average complexes were used to perform a new local docking using AutoDock.

#### 4.3.4 MD Simulations

The MD simulation of the complex was performed with the YASARA structure package according to our previously reported procedures (Floresta et al. 2019; Patamia et al. 2023).

## Conflicts of Interest

The authors declare no conflicts of interest.

## Supporting information


Data S1.

## Data Availability

The data that supports the findings of this study are available in the supporting information S1 material of this article.
