# Supplementary material for: Design, Synthesis, and Evaluation of Novel (−)‐cis‐N‐Normetazocine Derivatives: In Vitro and Molecular Modeling Insights
Source: Chem Biol Drug Des. 2024 Dec 26;104(6):e70037. doi: 10.1111/cbdd.70037 (PMC11671793; doi:10.1111/cbdd.70037)
Supplement: Supplementary file 1 — Data S1. [file CBDD-104-e70037-s001.docx]

**Supplementary Information**

**Design, synthesis, and evaluation of novel (–)-*cis*-*N*-normetazocine derivatives: *in vitro* and molecular modeling insights**

Giuliana Costanzo, Alessandro Coco, Giuseppe Cosentino, Vincenzo Patamia, Carmela Parenti, Emanuele Amata, Agostino Marrazzo, Antonio Rescifina, Lorella Pasquinucci*

*Corresponding authors: *E-mail address* [lpasquin@unict.it](mailto:lpasquin@unict.it) (L.P.)

**Table of contents**

| Graphs of MOR and KOR Ki values of compounds **3,4,7 and 8**. (Figure S1and S2) | S2 |
| --- | --- |
| ^1^H NMR and APT spectra of compounds **3** (Figure S3 and S4) | S3 |
| ^1^H NMR and APT spectra of compounds **4** (Figure S5 and S6) | S4 |
| ^1^H NMR and APT spectra of compounds **5** (Figure S7 and S8) | S5 |
| ^1^H NMR and APT spectra of compounds **6** (Figure S9 and S10) | S6 |
| ^1^H NMR and APT spectra of compounds **7** (Figure S11 and S12) | S7 |
| ^1^H NMR and APT spectra of compounds **8** (Figure S13 and S14) | S8 |
| Table S1: Elemental analysis data | S9 |

**
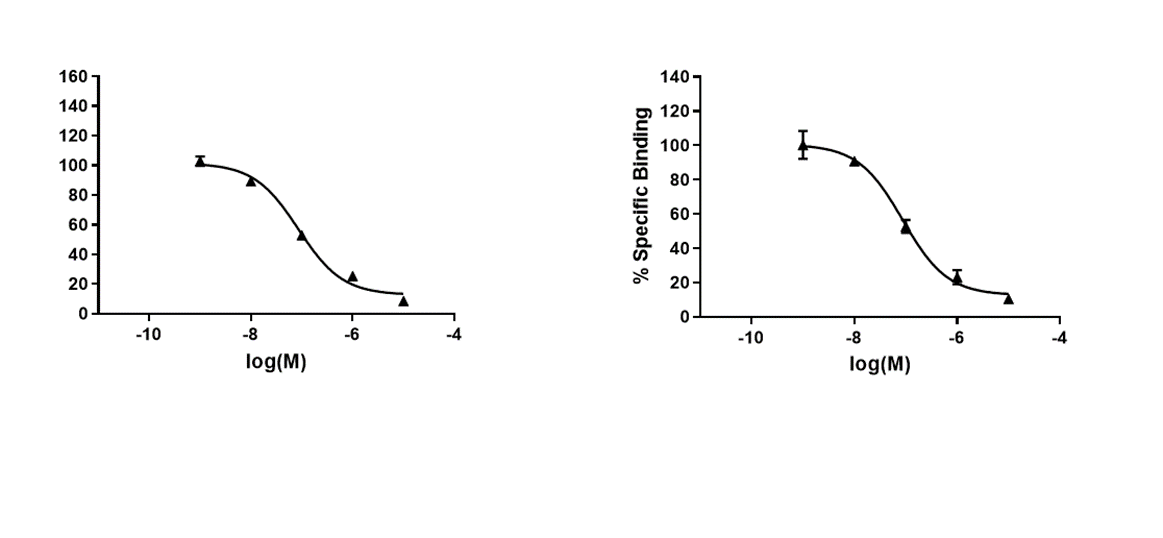
**

**Compound 4**

**Compound 3**

**
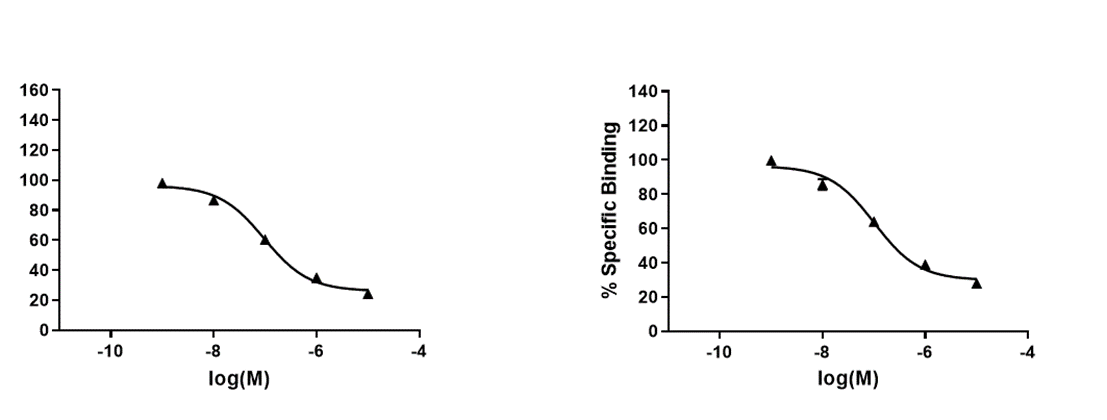
**

**Compound 8**

**Compound 7**

**FIGURE S1** Competitive inhibition of [^3^H]-DAMGO (1 nM) binding to rat brains membranes by unlabeled compounds **3**, **4** and **7**, **8** at 10^–9^–10^–5^ M concentration range. Data shown are expressed as percent-specific binding.

*
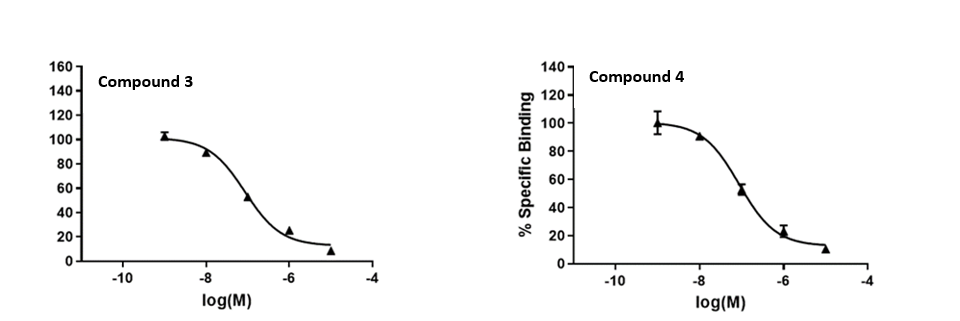
*

**FIGURE S2** Competitive inhibition of [^3^H]-U69,593 (1 nM) binding to guinea pig brains membranes by unlabeled compounds **3** and **4** at 10^–9^–10^–5^ M concentration range. Data shown are expressed as percent-specific binding.


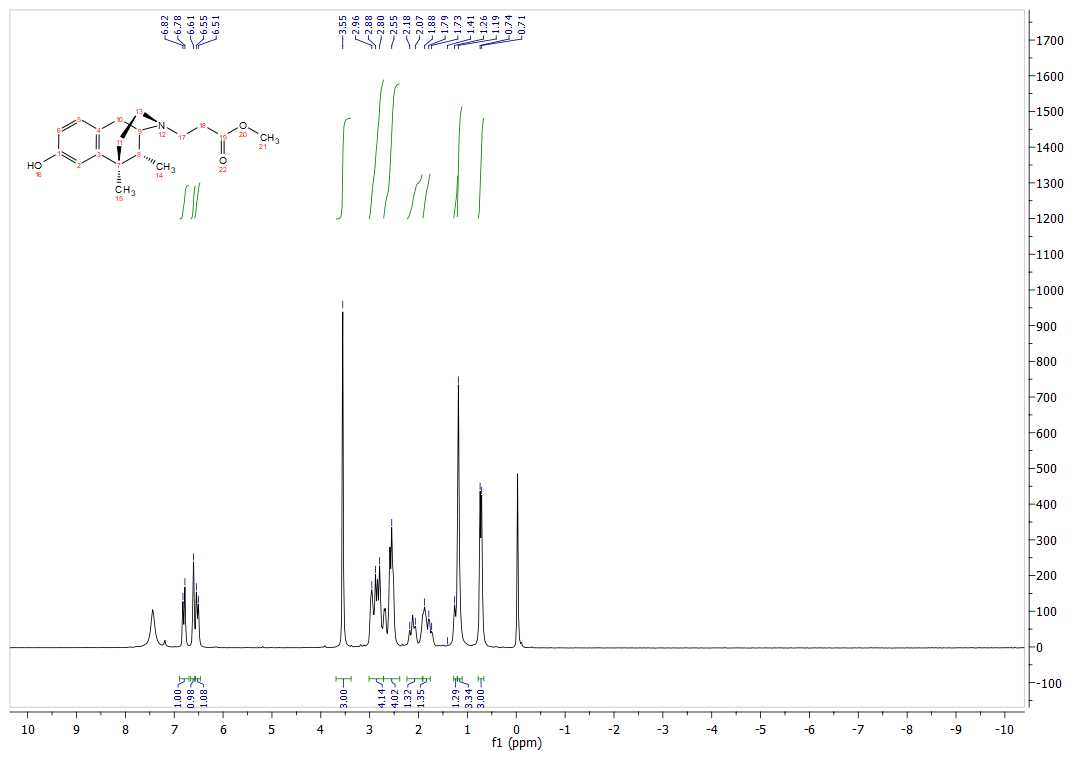


**FIGURE S3** ^1^H-NMR (200 MHz, CDCl_3_) spectrum of compound **3**.


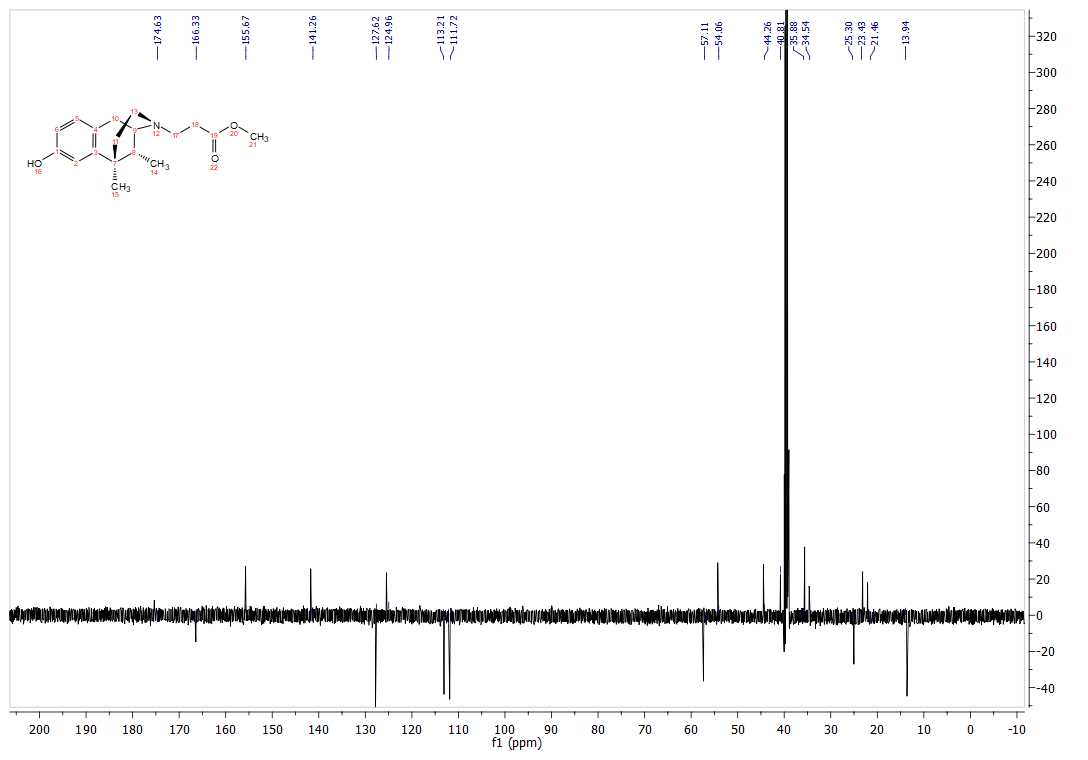


**FIGURE S4**  APT NMR (50 MHz, DMSO-*d_6_*) spectrum of compound **3.**


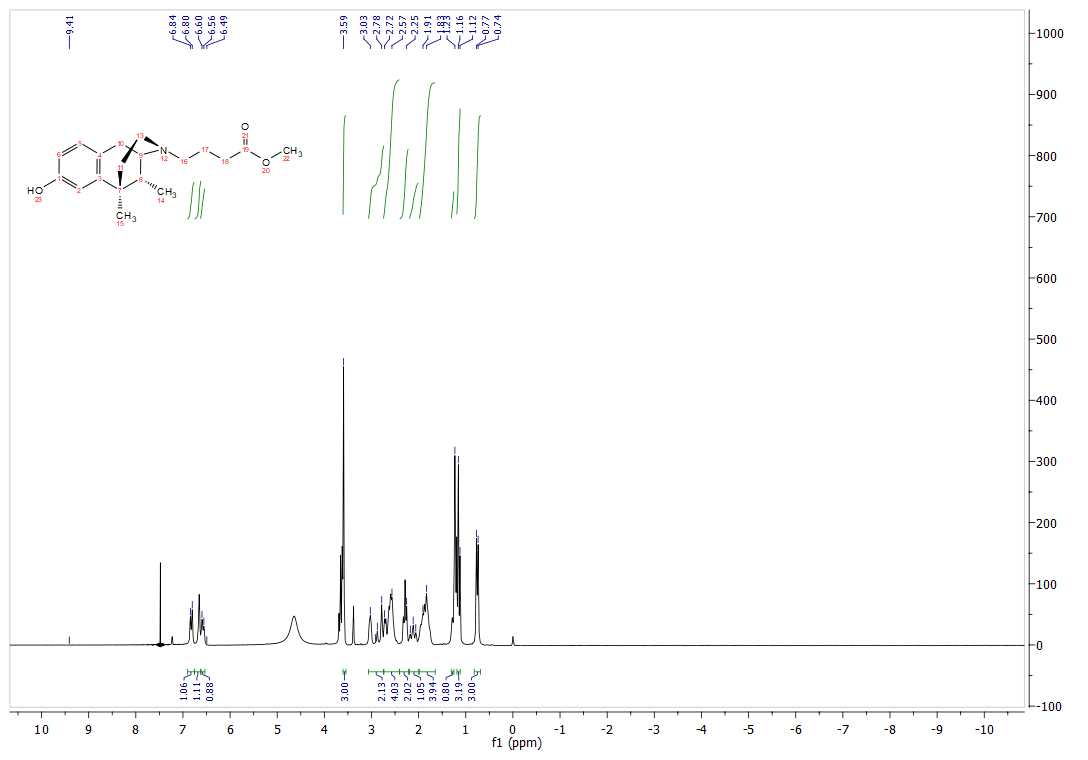


**FIGURE S5** ^1^H-NMR (200 MHz, CDCl_3_) spectrum of compound **4**.


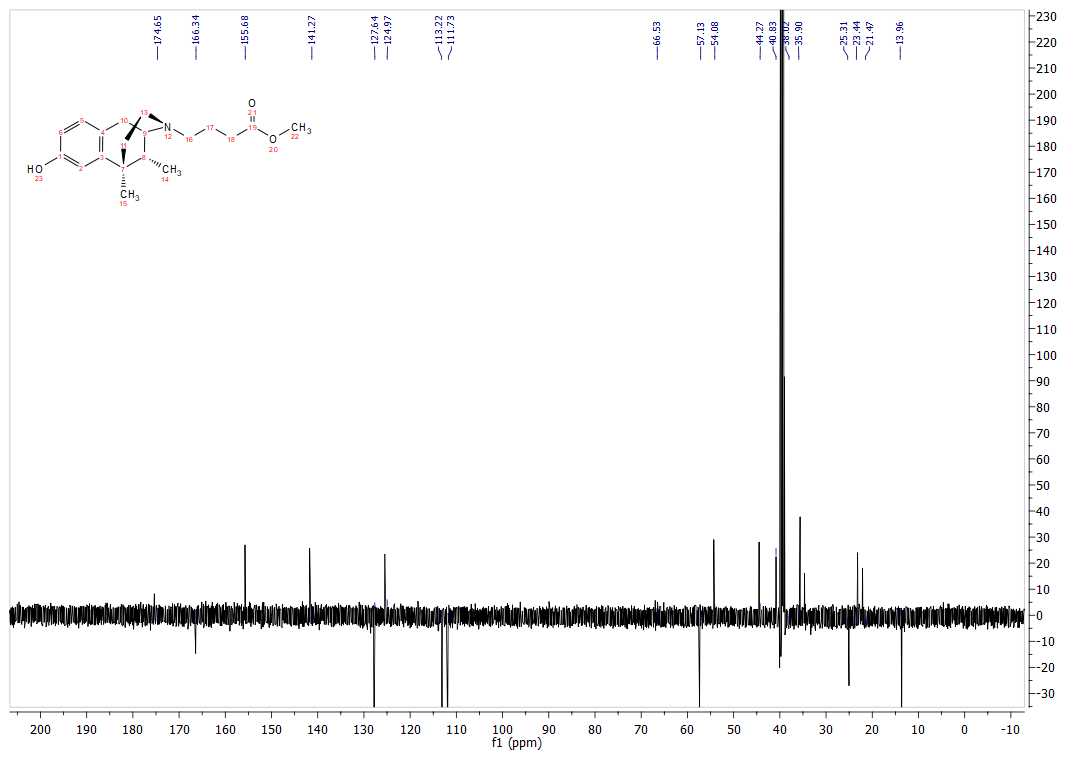


**FIGURE S6** APT (50 MHz, DMSO-*d_6_*) spectrum of compound **4**.


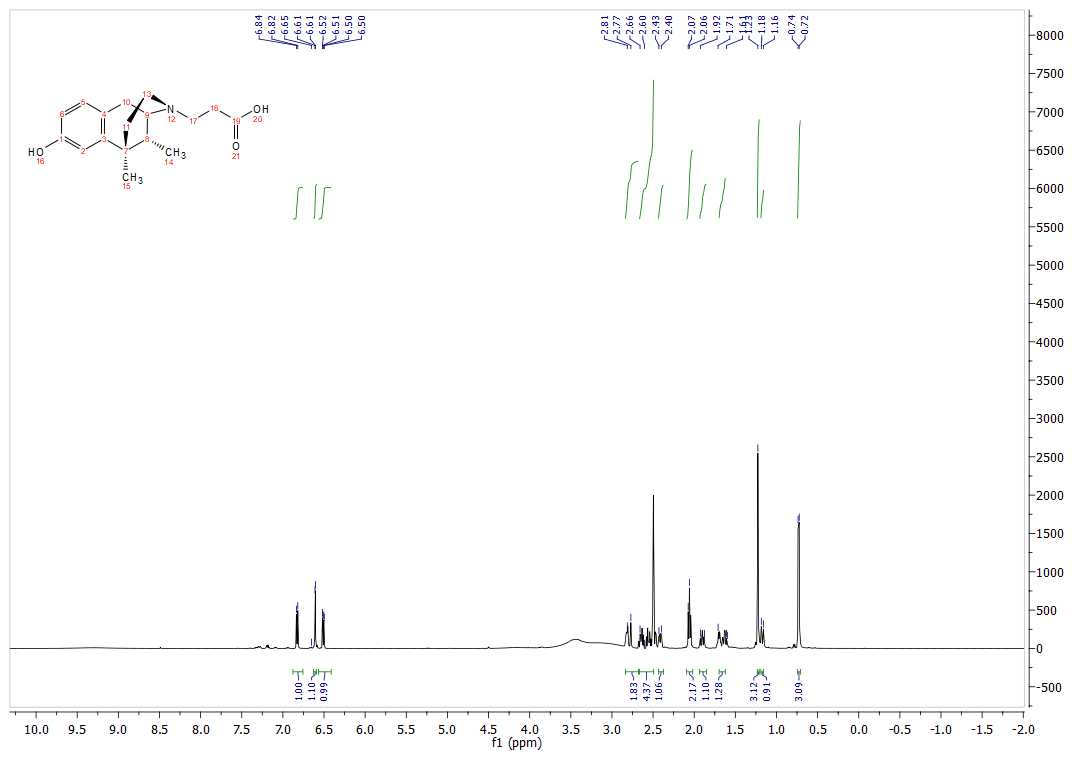


**FIGURE S7** ^1^H-NMR (500 MHz, DMSO-*d*_6_) spectrum of compound **5**.


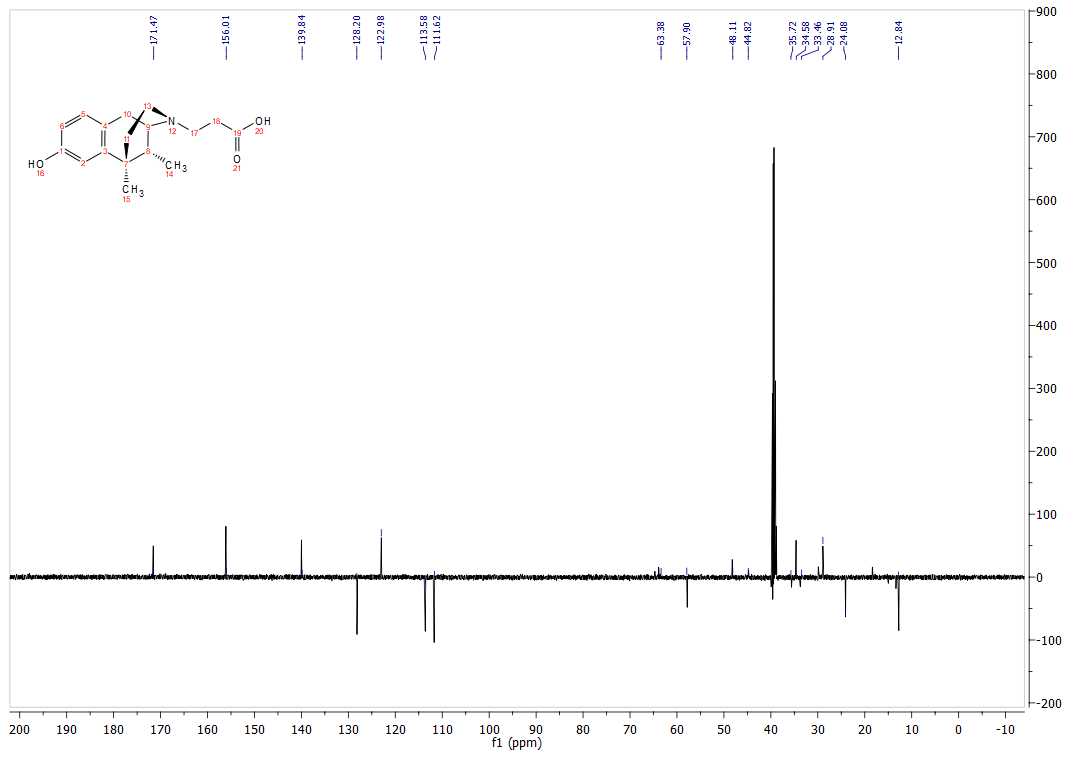


**FIGURE S8** APT NMR (125 MHz, DMSO-*d*_6_) spectrum of compound **5**.


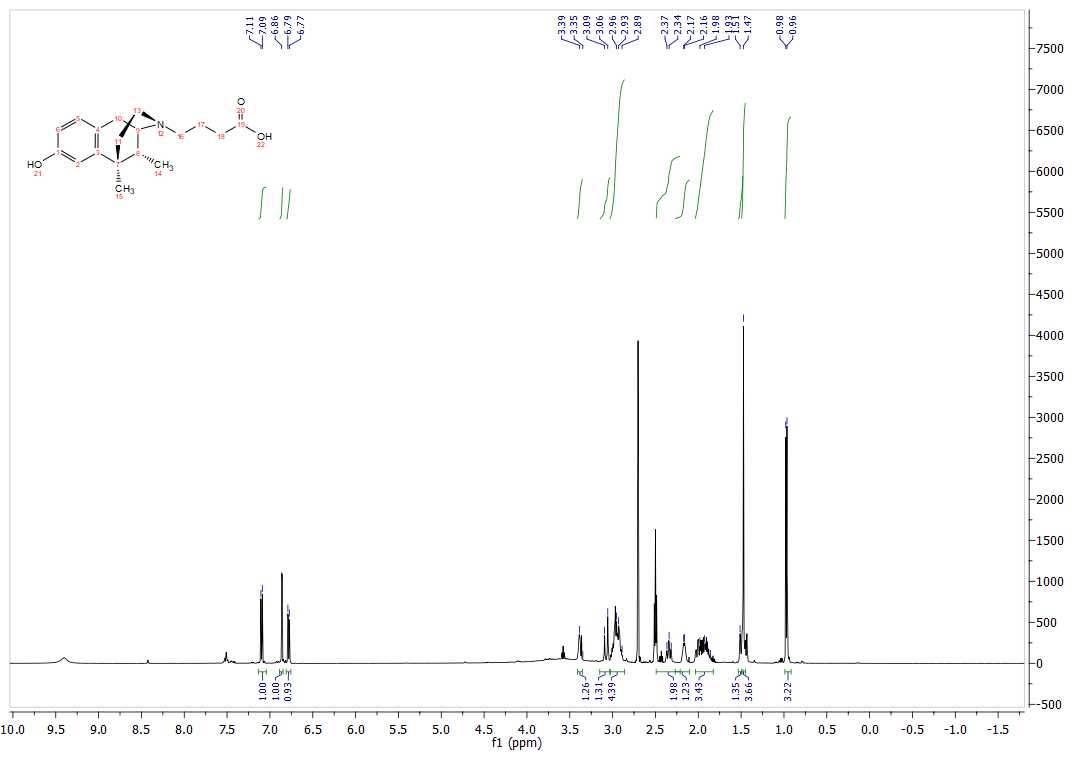


**FIGURE S9** ^1^H-NMR (500 MHz, DMSO-*d*_6_) spectrum of compound **6**.


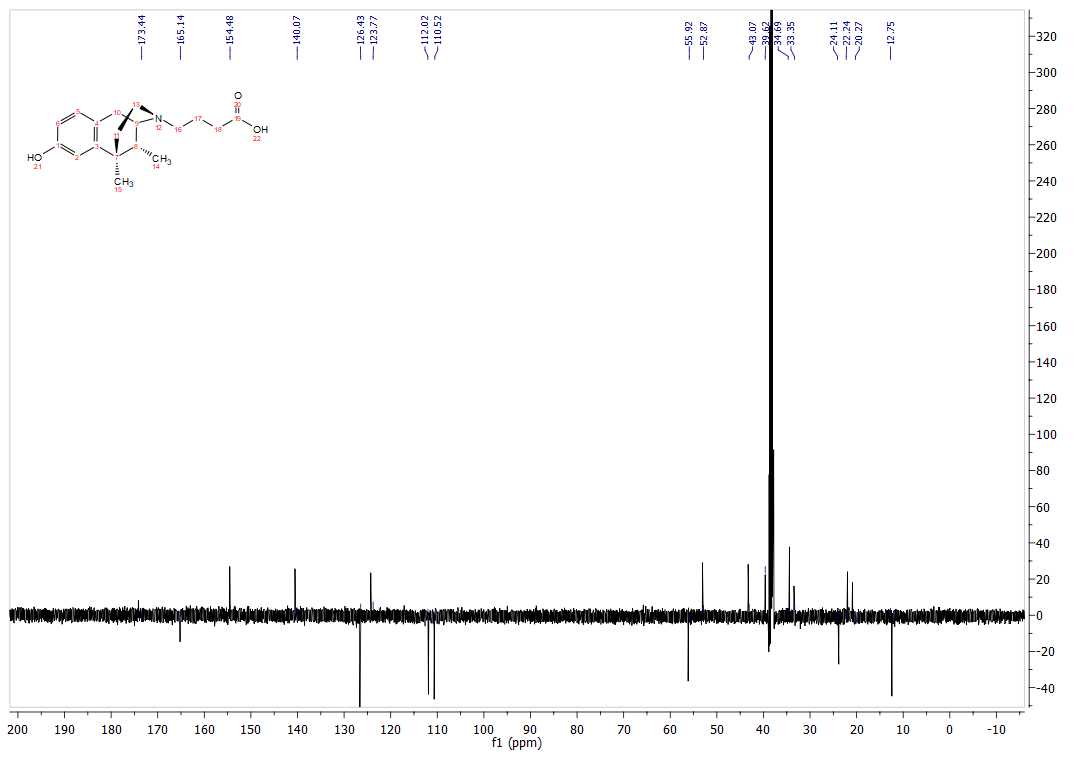


**FIGURE S10** APT NMR (125 MHz, DMSO-*d*_6_) spectrum of compound **6**.


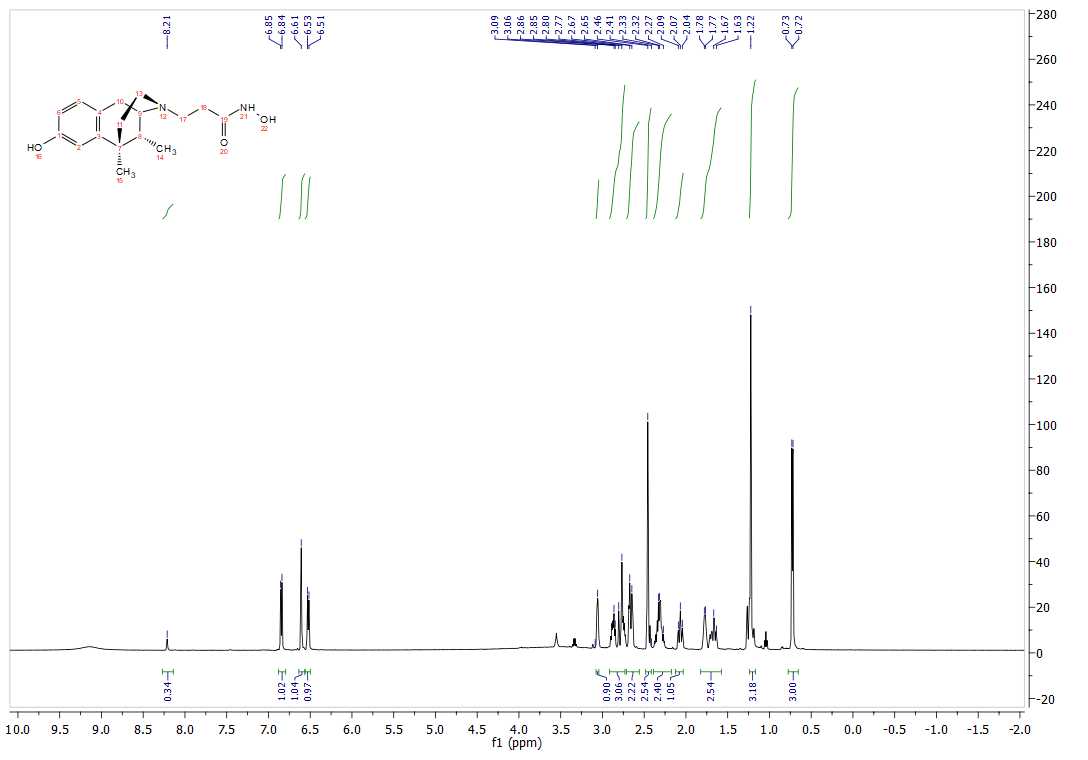


**FIGURE S11** ^1^H-NMR (500 MHz, DMSO-*d*_6_) spectrum of compound **7**.


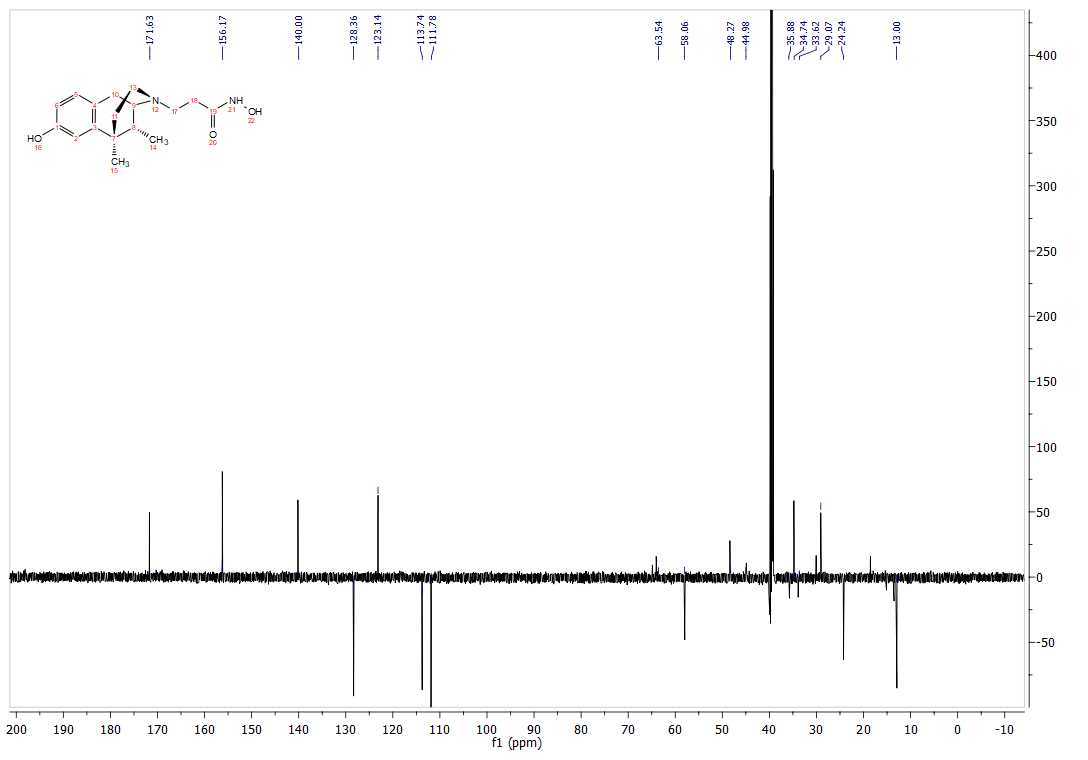


**FIGURE S12** APT (125 MHz, DMSO-*d*_6_) spectrum of compound **7**.


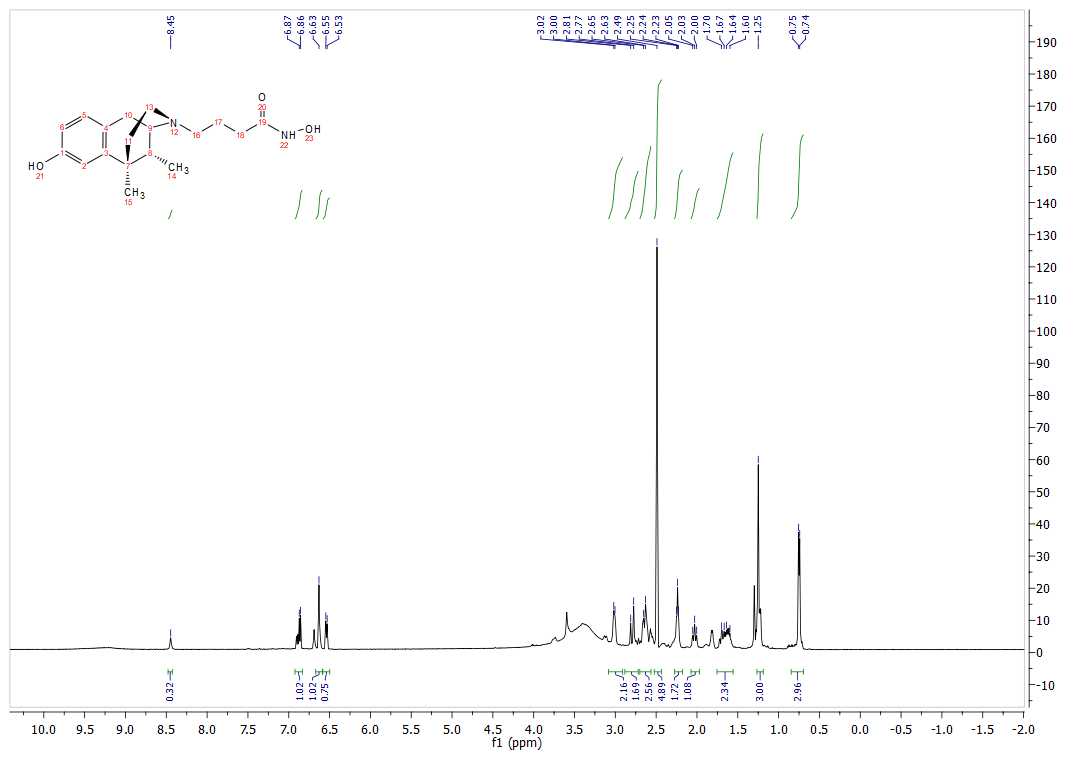


**FIGURE S13** ^1^H-NMR (500 MHz, DMSO-*d*_6_) spectrum of compound **8**.


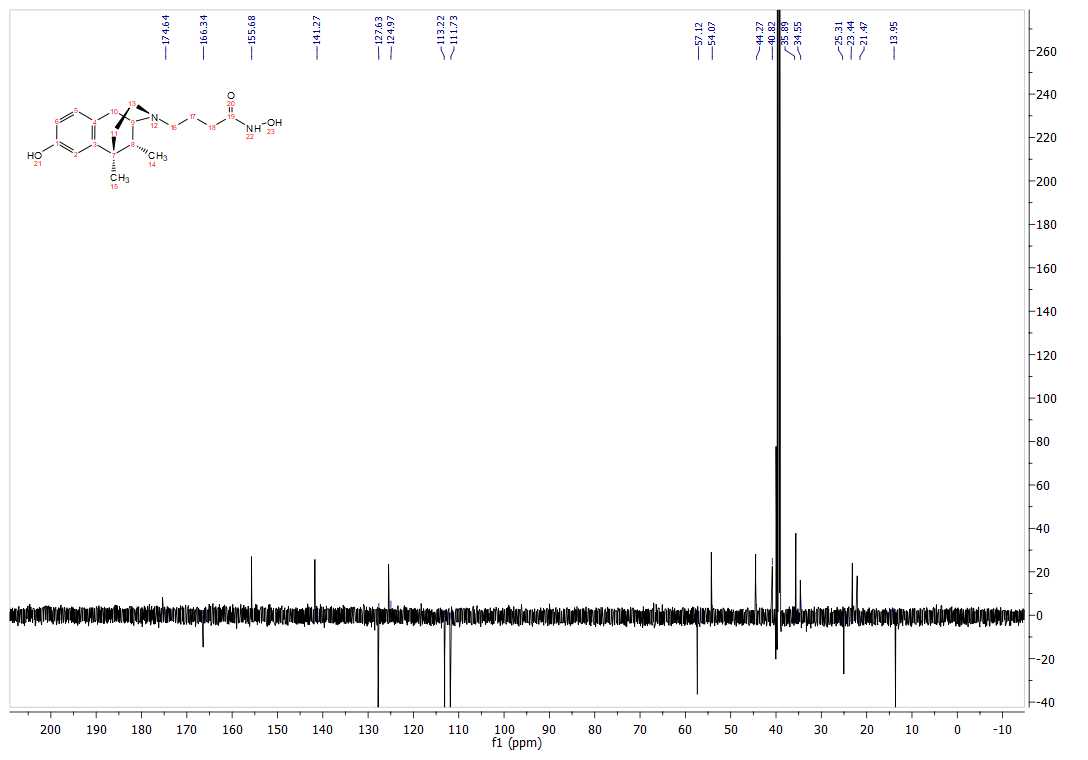


**FIGURE S14** APT (125 MHz, DMSO-*d*_6_) spectrum of compound **8**.

**TABLE S1** Elemental analysis data for compounds **3**−**8**.

|  |  |  | Calcd | | | Found | | |
| --- | --- | --- | --- | --- | --- | --- | --- | --- |
| **Compd** | **Formula** | **MW** | **C** | **H** | **N** | **C** | **H** | **N** |
| **3** | C_18_H_25_NO_3_ | 303.40 | 71.26 | 8.31 | 4.62 | 71.35 | 8.02 | 4.51 |
| **4** | C_19_H_27_NO_3_ | 317.43 | 71.89 | 8.57 | 4.41 | 71.64 | 8.29 | 4.32 |
| **5** | C_17_H_23_NO_3_ | 289.17 | 70.56 | 8.01 | 4.84 | 70.82 | 8.28 | 4.61 |
| **6** | C_18_H_25_NO_3_ | 303.40 | 71.26 | 8.31 | 4.62 | 71.19 | 8.40 | 4.50 |
| **7** | C_17_H_24_N_2_O_3_ | 304.18 | 67.08 | 7.95 | 9.20 | 67.10 | 8.03 | 9.37 |
| **8** | C_18_H_26_N_2_O_3_ | 318.19 | 67.90 | 8.23 | 8.80 | 67.67 | 8.47 | 8.85 |
